# Photochemical Deracemization of a Medicinally‐Relevant Benzopyran using an Oscillatory Flow Reactor

**DOI:** 10.1002/chem.202200741

**Published:** 2022-04-05

**Authors:** Jason D. Williams, Peter Pöchlauer, Yoshiyuki Okumura, Yukari Inami, C. Oliver Kappe

**Affiliations:** ^1^ Center for Continuous Flow Synthesis and Processing (CCFLOW) Research Center Pharmaceutical Engineering GmbH (RCPE) Inffeldgasse 13 8010 Graz Austria; ^2^ Institute of Chemistry University of Graz NAWI Graz Heinrichstrasse 28 8010 Graz Austria; ^3^ Thermo Fisher Scientific Linz St.-Peter-Straße 25 4020 Linz Austria; ^4^ R&D and Business Promotion AskAt Inc. 2F Dai-Tokai Building 3-22-8 Meieki Nakamura-ku Nagoya Aichi 450-0002 Japan

**Keywords:** chiral resolution, crystallization-induced diastereomer transformation, deracemization, flow chemistry, photochemistry

## Abstract

Dynamic deracemization processes, such as crystallization‐induced diastereomer transformations (CIDTs), offer the opportunity to combine racemization and resolution processes, to provide high yields of enantiomerically pure compounds. To date, few of these processes have incorporated photochemical racemization. By combining batch crystallization with a flow photoreactor for efficient irradiation, it is possible to perform such deracemization in an effective, scalable and high yielding manner. After applying design of experiment (DoE) principles and mathematical modelling, the most efficient parameter set could be identified, leading to excellent results in just 4 h reaction time: isolated yield of 82 % and assay *ee* of 96 %. Such photochemical racemization methods can serve to open new avenues for preparation of enantiomerically pure functional molecules on both small and industrially‐relevant scales.

## Graphical abstract

**Photo‐transformation**: Unlike classical enantiomeric resolution methods, crystallization‐induced diastereomer transformations (CIDTs) allow full conversion to the desired enantiomer. Using photochemical racemization for a CIDT has not before been published in the scientific literature. We report the use of an oscillatory flow reactor for efficient irradiation, allowing high yield and >95 % *ee* to be achieved in a reaction time of just a few hours.

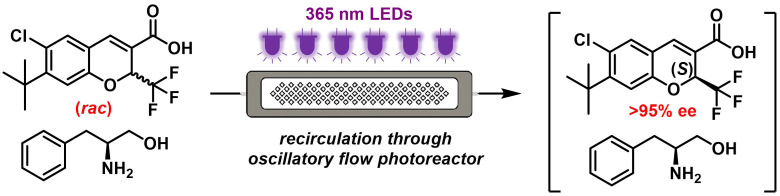

The preparation of enantiomerically pure compounds has provided a challenge within the pharmaceutical industry (amongst others) over the past half century. Stereogenic centers can be problematic in organic synthesis, particularly for medicinally‐relevant molecules, whose different enantiomers can have greatly varied biological properties.^[1]^ In particular, chiral compound AAT‐076 (Scheme 1) has been demonstrated to be a potent COX‐2 (cyclooxygenase‐2) inhibitor and has been investigated as a treatment for inflammation and pain.^[2]^

**Scheme 1:**
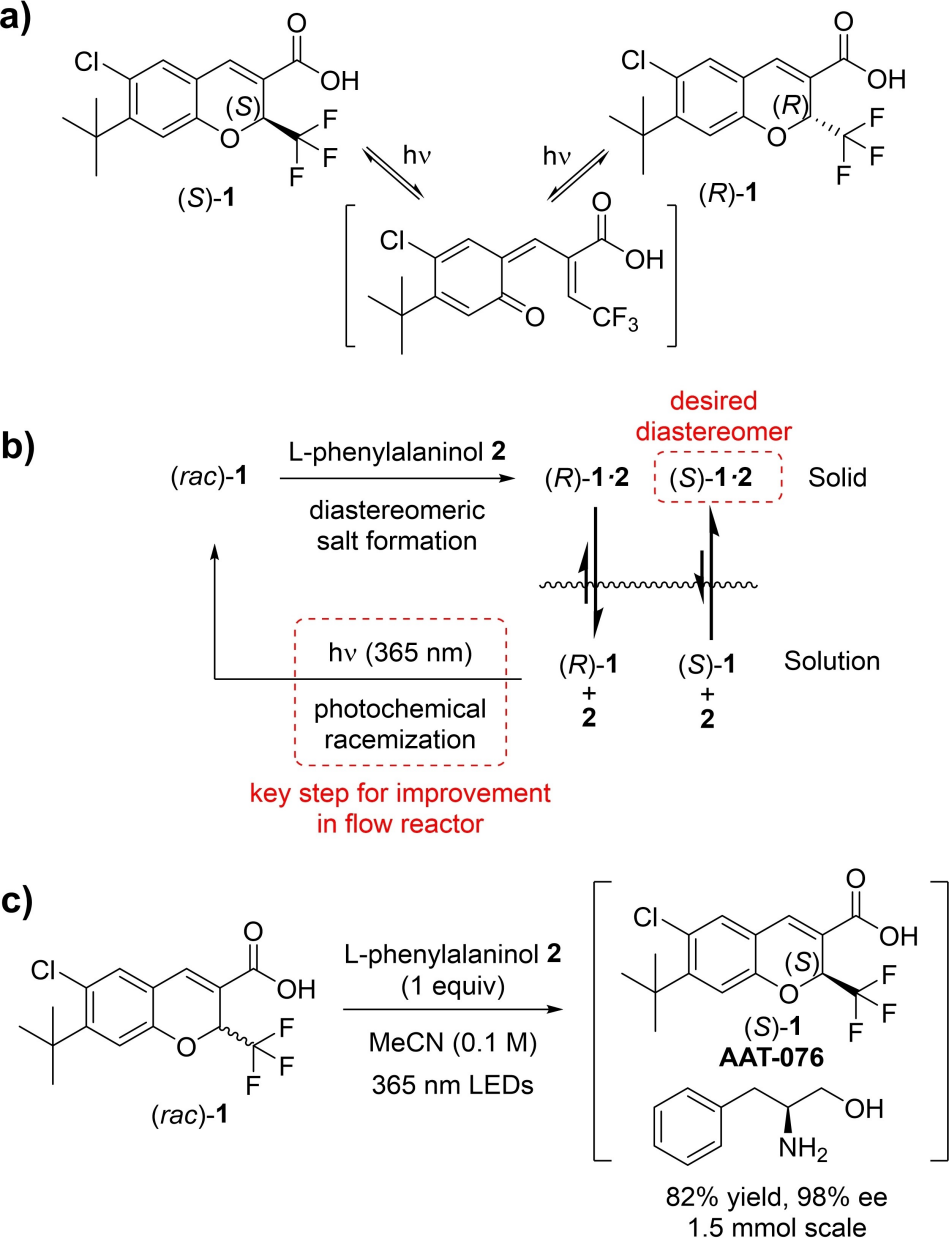
**a)** Previously proposed mechanism of photochemical racemization, via retro‐Claisen.^[10]^ **b)** A schematic view of the CIDT process described in this work. l‐Phenylalaninol (**2**) acts as a resolving agent, causing the desired enantiomer to favorably precipitate as its less soluble diastereomeric salt. In the meantime, the soluble fraction of (*R*)‐**1** and (*S*)‐**1** are continuously photochemically racemized, leading to further enriched solid, (*S*)‐**1⋅2**. **c)** Structure of racemic API (*rac*)‐**1** and deracemization process (this work), leading to enantiomerically pure material as a diastereomeric salt.

Although there has been a significant increase in synthetic methods available for the introduction of stereocenters,^[3]^ many compounds still rely upon separation/resolution to produce optically pure material.^[4]^ Indeed, AAT‐076 has previously been prepared using chiral chromatography,^[5]^ or resolution by selective diastereomer crystallization with l‐phenylalaninol.^[6]^ Such classical separation methods are inherently wasteful, yet there are numerous situations where active racemization can be combined with diastereoselective crystallization, which allows enantiomeric enhancement without any requirement for loss of material. This method, known as crystallization‐induced diastereomer transformation (CIDT), has been well‐utilized in a large number of applications.^[7]^ However, such an approach requires an equilibrating stereocenter, limiting the number of compatible molecules, most often to those with a stereocenter adjacent to a carbonyl group.

An additional possible mechanism for stereocenter equilibration is via a light‐induced transformation. This is more commonly utilized for E/Z‐type isomerization,^[8]^ but some racemization examples are also known.^[9]^ Specifically, it has been reported that benzopyran derivatives, such as AAT‐076 ((*S*)‐**1**), can undergo racemization via a retro‐Claisen pathway (Scheme 1a).^[10]^

For such a CIDT process to be effective and scalable, efficient racemization is a prerequisite. This may be expedited using flow reactor technology for improved light intensity and irradiation homogeneity.^[11]^ Recent reports have seen success utilizing flow reactors to recirculate a reaction mixture, spatially separating the racemization and crystallization aspects of CIDT processes.^[12]^ To our knowledge, though, this approach has not before been described for CIDT in which racemization is photochemically induced (Scheme 1b). Herein we present CIDT of (*rac*)‐**1**, to its desired diastereomeric salt (*S*)‐**1⋅2** in high yield (>80 %) and *ee* (>95 %). By recirculating through a flow photoreactor, the efficiency of the photochemical racemization step is significantly improved, whilst maintaining effective crystallization in a batch vessel.

By combining the previously reported l‐phenylalaninol resolution process^[6]^ with UVA irradiation, it is possible to convert (*rac*)‐**1** to the desired (*S*)‐**1**, for isolation as the diastereomeric salt in high yield and good *ee* (Scheme 1c).^[13]^ Such a process shows great promise for the production of enantiopure AAT‐076, compared to previous classical resolution approach (30 % yield reported).^[6]^ For small scale processing, up to a few grams, this procedure is a good option, but provides no suitable scale‐up route and already shows very different behavior when operated on 15 mmol scale. Accordingly, a study was commenced to improve its efficiency and scalability.

Initial analysis considered the extinction coefficient of the substrate, which was found experimentally to be 3728 M^−1^ cm^−1^ at the desired irradiation wavelength (365 nm). This value is exceptionally high for a directly irradiated species, at the desired concentration of 0.1 M. Indeed, it can be calculated that >95 % of light is absorbed within just 0.04 mm distance from the reactor vessel wall (see Supporting Information for details of UV/vis measurements and Beer‐Lambert calculations). Accordingly, the efficiency of such a transformation will decrease exponentially with increasing reaction scale, as the irradiated surface area to volume ratio decreases.

A useful solution to this can be to use a flow reactor to efficiently irradiate the resolution mixture.^[11]^ This will drastically increase the irradiated surface area and can be scaled in a more linear fashion, particularly when considering the concept of “numbering up” (i. e. using multiple flow reactors in parallel).^[14]^ In such a way, the irradiated surface area can remain high, guaranteeing a constant rate of racemization, independent of reaction scale.

In order to examine this effect experimentally, the pure (*R*)‐enantiomer (*R*)‐**1** was racemized using UVA irradiation (365 nm LEDs) in two different reaction setups. On small scale (1.5 mmol, 15 mL), a good level of racemization was achieved in 1 h. However, upon increasing the scale (15 mmol, 150 mL), almost 4 h was required to reach the same level. To improve this racemization rate, a comparison was made against recirculation through a high surface area flow reactor (Figure 1). The batch setup featured a round‐bottomed flask with external irradiation, whereas flow irradiation was achieved by recirculated through a 15 mL flow reactor (HANU HX 15, Creaflow, irradiated surface area=66 cm^2^, channel depth=2 mm).^[15]^

**Figure 1:**
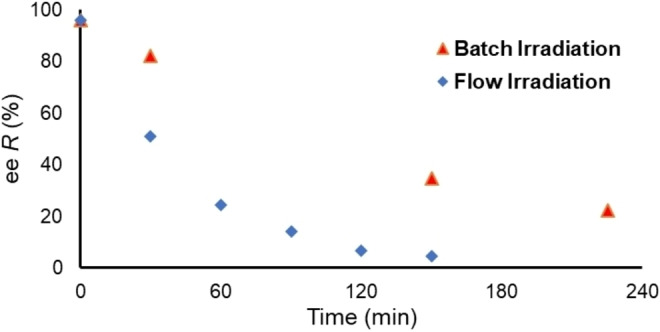
Comparison of time required to racemize pure (*R*)‐**1** in a classical batch setup, versus recirculation through a flow reactor. Chiral purity determined at each time point by chiral HPLC. Reactions were performed on 15 mmol scale.

As expected, racemization of the enantiomerically pure material (*R*)‐**1** to (*rac*)‐**1** occurred significantly faster within the flow reactor. This rate of racemization was anticipated to be the limiting factor in the CIDT resolution process for production of chirally pure (*S*)‐**1**, therefore maximizing its rate is vital for effective processing.

Whilst the batch reaction contains a slurry of the diastereomeric salt, recirculation through a flow reactor also presents an opportunity to filter the irradiated fraction of the resolution mixture. This will further enhance light penetration, for improved racemization efficiency. Accordingly the flow reactor was set up with a filter at its inlet, confining solids to the stirred batch vessel. An oscillatory pump^[16]^ was also used, in order to ensure that any solids formed within the flow reactor would be effectively suspended and not settle or cause blockages (Figure 2a).

**Figure 2:**
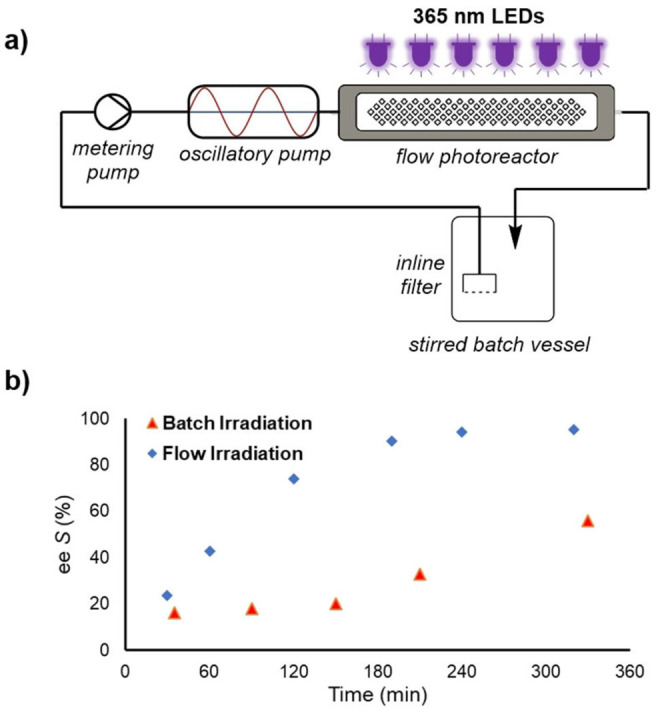
**a)** Schematic view of the reactor setup used for CIDT of (*rac*)‐**1**, based on a batch vessel with an inlet filter, recirculating through an irradiated flow reactor. **b)** Comparison of resolution efficiency in batch vs. recirculated flow reactor, showing the evolution of *ee* of the solid phase over time.

In comparison to the standard batch setup, this resulted in a significantly faster resolution process, whereby the *ee* of the solid phase reached >95 % within ∼5 h, whilst the batch equivalent reached only 56 % *ee* in the same reaction time. Since the rate of CIDT follows an exponential function, 23 h was required in batch to reach acceptable *ee* (>95 %). After the initial success achieved with the flow irradiation setup, additional experiments were performed to further improve the rate of CIDT and to improve the isolation procedure for the desired diastereomeric salt.

A CIDT attempt was also made with a modified reactor setup, whereby the inlet filter in the holdup vessel was removed. Due to the nature of the reactor and the use of an oscillatory pump, it was feasible to convey the slurry directly through the reactor. However, the resolution process was observed to be slower, likely because of poorer irradiation of the slurry, compared with the monophasic reaction mixture (see Supporting Information for details).

Additional insight to the state of the process could be drawn, by also analyzing the liquid phase throughout the course of the reaction. Whilst the resolution process was ongoing, an excess of ∼40 % *(R)‐* **1** was present in the liquor, confirming that the photochemical step is, in this case, rate‐limiting. As the resolution process came to an end, the liquid phase was observed to tend toward a racemic mixture, implying that no further selective crystallization will occur. By adding the amine **2** in portions, the excess of (*R*)‐**1** in the liquors was lower, whilst maintaining a fast CIDT rate. This implies that improved results could be achieved by modulating this addition rate (see Supporting Information for further details and data).

With the aim of optimizing this process for further scale‐up and production applications, a design of experiments (DoE) study was carried out.^[17]^ By using this approach, as opposed to classical one‐factor‐at‐a‐time optimization, it was envisaged that a more thorough understanding of the relationship between reaction parameters (factors) and outcomes (responses) could be drawn. Furthermore, predictive mathematical models can be parameterized, facilitating process predictions toward optimal conditions.

Based on initial experiments, four factors were identified to be of interest: 1) light intensity; 2) amine **2** equivalents; 3) amine **2** addition rate; 4) reaction scale. To examine the influence of these factors, and their interactions with one another, a full factorial design was utilized, consisting of 16 experiments and two center‐points (as a measure of reproducibility). During these experiments, 6 responses were measured: yield and *ee* of isolated material, as well as *ee* measured at 0.5, 2, 4 and 6 h. These different time points were anticipated to serve as a view of the *ee* evolution over time, allowing the fastest CIDT conditions to be identified.

Statistical analysis of the results of this study showed that, surprisingly, varying the light intensity (from 6–10, where 10 is the maximum) had no effect on any of the measured variables. This unexpected outcome can be rationalized by the previously discussed poor light penetration. It follows that the more important parameter in this example is the irradiated surface area, which can be maximized by careful reactor design and a sound scale‐up strategy.

By far the most influential factors were found to be the equivalents and addition rate of l‐phenylalaninol (**2**). The surface plots of isolated yield and *ee* at 4 h (Figure 3a and Figure 3b, respectively) against these two factors contain several interesting features. Amine **2** (equiv, shown on the x axis) shows significant curvature, with both responses at a maximum in the center (or slightly lower, around 1.10, for *ee* at 4 h). The addition rate (y axis) shows a more complex picture, since higher yield is favored by more amine portions (i. e. extended addition period), whilst the *ee* at 4 h is highest with faster amine addition. See the Supporting Information for all DoE results and developed models.

**Figure 3:**
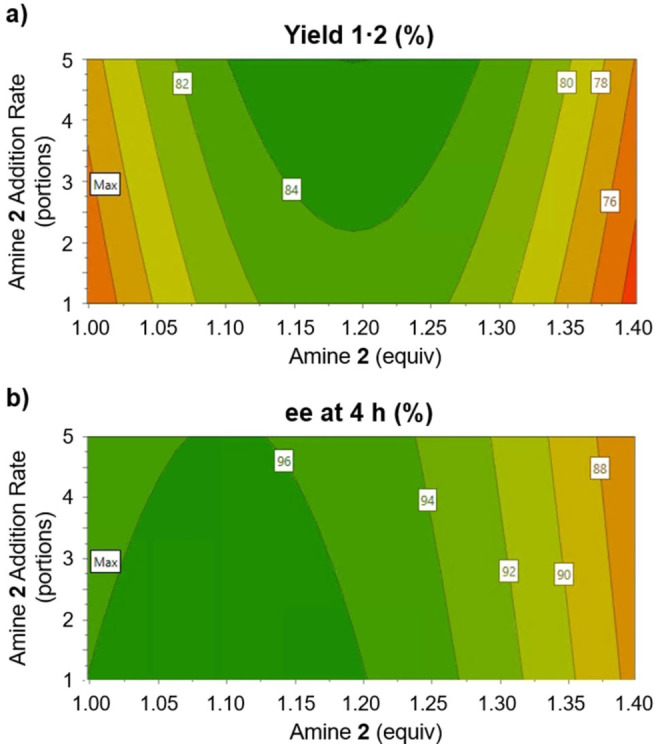
Surfaces generated from reaction models, depicting **a)** Isolated yield of the desired diastereomeric intermediate after 6 h reaction; **b)** *ee* of the solid phase, measured after 4 h. General conditions: 15 mmol scale, light intensity=6 (of 10).

External validation of the models with a separate experiment was performed, showing responses within the interquartile prediction ranges (see Supporting Information). Therefore, using these models as a guide, reaction conditions were chosen to provide the best tradeoff of yield vs. *ee* within 4 h reaction time. An experiment was carried out with the suggested factor settings (Scheme 2): light intensity=6; 1.15 equiv **2**; moderate addition rate (3 portions); 20 mmol scale; 4 h reaction time. Despite problems with batch reactor agitation, the reaction yielded 7.97 g (82 % yield) of the desired salt (*S*)‐**1⋅2**, and the material sampled at 4 h was found to have 96 % *ee*. It is envisaged that the *ee* will be further upgraded in a crystallization following the acidification of (*S*)‐**1⋅2**, to remove the amine counterion.

**Scheme 2:**
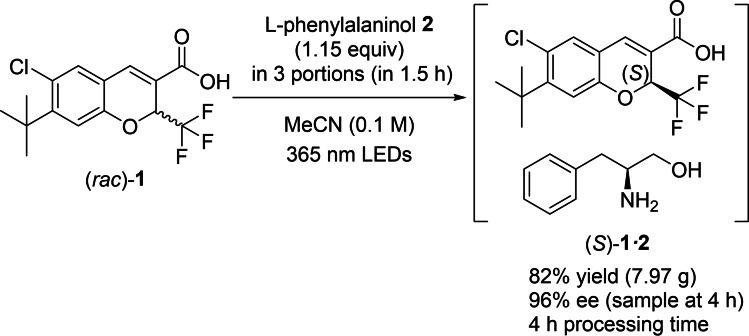
Final conditions used to produce (*S*)‐**1** as a diastereomeric salt with l‐phenylalaninol (**2**) in a recirculated flow setup.

In conclusion, we have described the first photochemical CIDT process using recirculation through a flow photoreactor. The process is capable of achieving a high yield (∼80 %) and high *ee* (>95 %) in a short reaction time of only 4 h. Through a DoE study, the influence of reaction parameters have been well understood, resulting in mathematical models to target the most suitable conditions. The identified conditions are expected to provide an industrially‐relevant process for production of enantiopure AAT‐076, with an effective scale‐up route identified. This work will serve to open the door to related photochemical CIDT processes for other APIs and functional molecules, whereby chiral purity will no longer serve as a barrier to efficient synthesis routes.

## Conflict of interest

The authors declare no conflict of interest.

## Supporting information

As a service to our authors and readers, this journal provides supporting information supplied by the authors. Such materials are peer reviewed and may be re‐organized for online delivery, but are not copy‐edited or typeset. Technical support issues arising from supporting information (other than missing files) should be addressed to the authors.

Supporting Information

## Data Availability

The data that support the findings of this study are available in the supplementary material of this article.
