# Supplementary material for: Photochemical Deracemization of a Medicinally‐Relevant Benzopyran using an Oscillatory Flow Reactor
Source: Chemistry. 2022 Apr 5;28(29):e202200741. doi: 10.1002/chem.202200741 (PMC9321886; doi:10.1002/chem.202200741)
Supplement: Supplementary file 1 — Supporting Information [file CHEM-28-0-s001.pdf]

Chemistry–A European Journal

Supporting Information

Photochemical Deracemization of a Medicinally-Relevant Benzopyran using an Oscillatory Flow Reactor

Jason D. Williams, Peter Pöchlauer, Yoshiyuki Okumura, Yukari Inami, and C. Oliver Kappe*

Table of Contents

1. Experimental Details 1
1.1. Materials 1
1.2. Analytical Methods 1
1.3. Batch Reaction Setup 4
1.4. Batch Reaction Procedure (15 mmol) 4
1.5. Flow Reaction Setup 5
1.6. Representative Flow Reaction Procedure 8
1.7. Sampling Procedure and Sample Preparation 9
2. UV/Vis Analysis of Starting Material 10
3. ee Data of Liquid Phase 12
4. DoE Data 13
4.8. DoE Setup 13
4.9. Analysis of DoE Results 16
4.9.1. Yield 17
4.9.2. ee Isolated 20
4.9.3. ee 0.5 h 23
4.9.4. ee 2 h 26
4.9.5. ee 4 h 29
4.9.6. ee 6 h 32
4.10. Validation Experiment 35
4.11. Final Experiment 37
5. Characterization Data 40
6. References 41
7. NMR Spectra 42

1. Experimental Details

1.1. Materials

R-7-(*tert*-butyl)-6-chloro-2-(trifluoromethyl)-2*H*-chromene-3-carboxylic acid (*R*)-**1** (SC75417) was supplied by AskAt.

S-7-(*tert*-butyl)-6-chloro-2-(trifluoromethyl)-2*H*-chromene-3-carboxylic acid (*S*)-**1** (SC75416) was supplied by AskAt.

MeCN was purchased from VWR (HPLC grade, ≥99.95% purity, Article# 83639.320)

L-Phenylalaninol was purchased from abcr (98% purity, Article# AB168441).

1.2. Analytical Methods

Chiral HPLC:

Measurements of chiral purity were made using a Shimadzu HPLC system (DGU-14A degasser, SCL-10A VP system controller, SPD-10 UV-VIS detector, LC-20AT pumps)

Column: Chiralpak® IA (4.6 × 250 mm, 5µm)

Flow rate: 0.7 mL/min

Mobile phase: Heptane/EtOH/trifluoroacetic acid (90/10/0.2)

Injection volume: 1 µL

Detection wavelength: 342 nm

Retention times: *R*-isomer 6.23 min; *S*-isomer 7.20 min

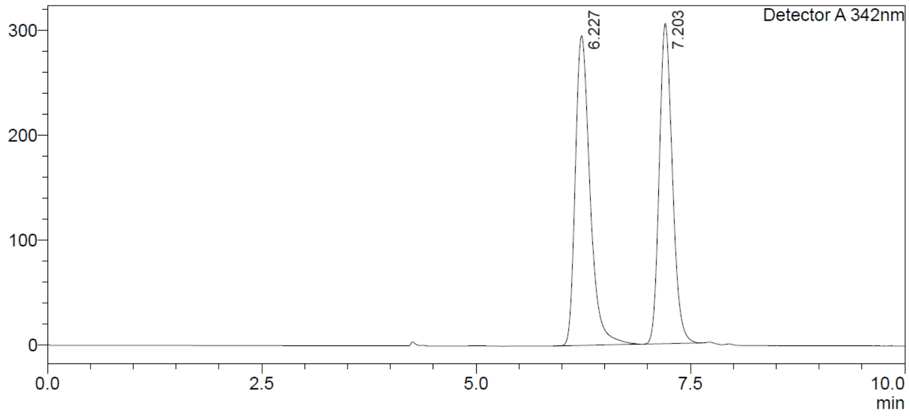

Figure S1. An example chiral HPLC chromatogram, showing a racemic mixture of both enantiomers (*rac*)-**1**.

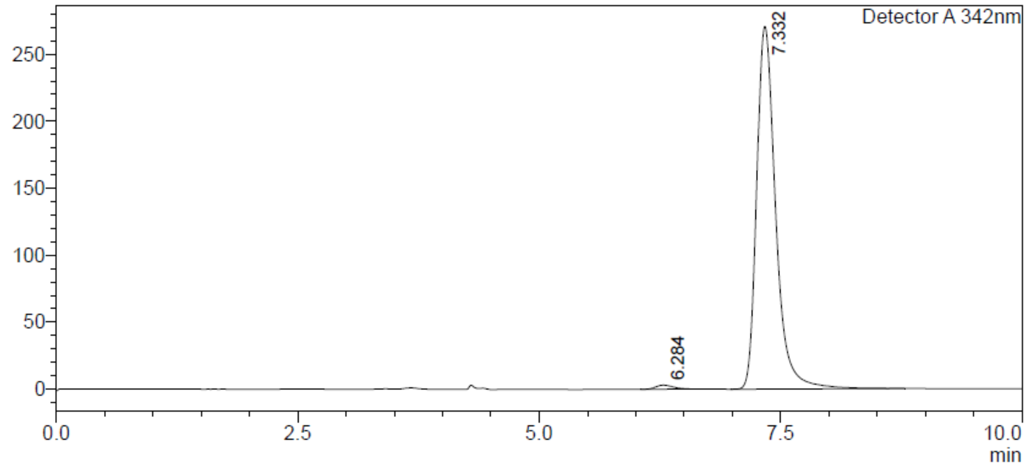

Figure S2. An example chiral HPLC chromatogram, showing a sample of solid (*S*)-**1·2** with 98% ee.

Standard HPLC:

Analytical HPLC (Shimadzu LC20) analysis was carried out on a C18 reversed-phase analytical column (150 mm × 4.6 mm, particle size 5 µm) at 37 °C using mobile phases A (90:10 v/v water/acetonitrile + 0.1% TFA) and B (MeCN +0.1% TFA) at a flow rate of 1.5 mL/min. The following gradient was applied: start at 30% solvent B, increase to 100% solvent B over 13 min.

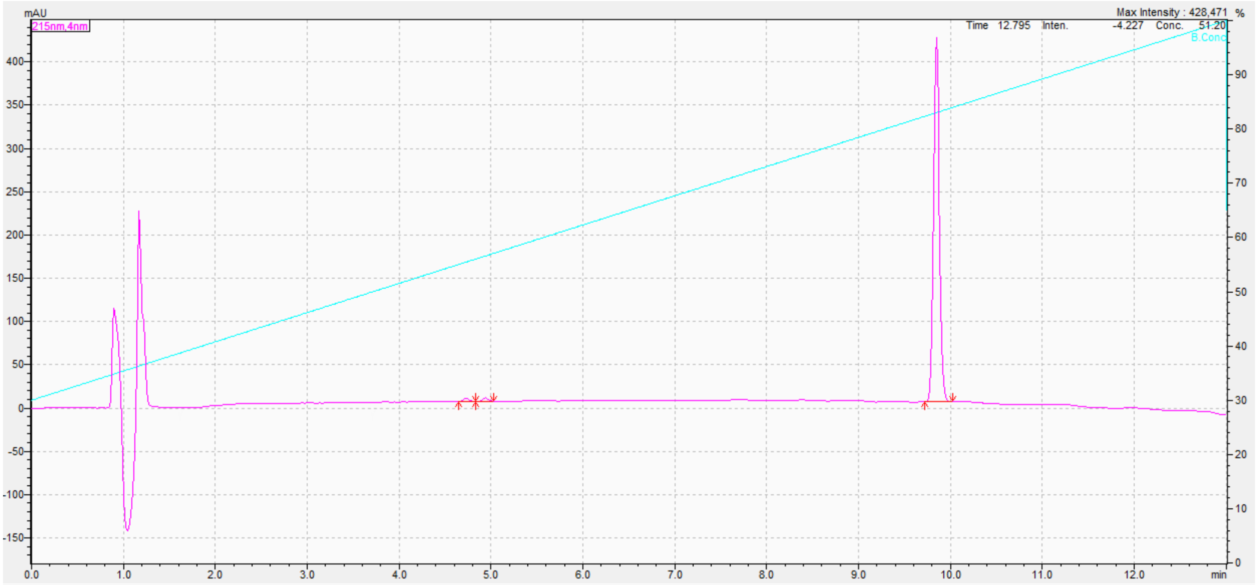

Figure S3. An example standard HPLC chromatogram of the solid (*S*)-**1·2** at 215 nm.

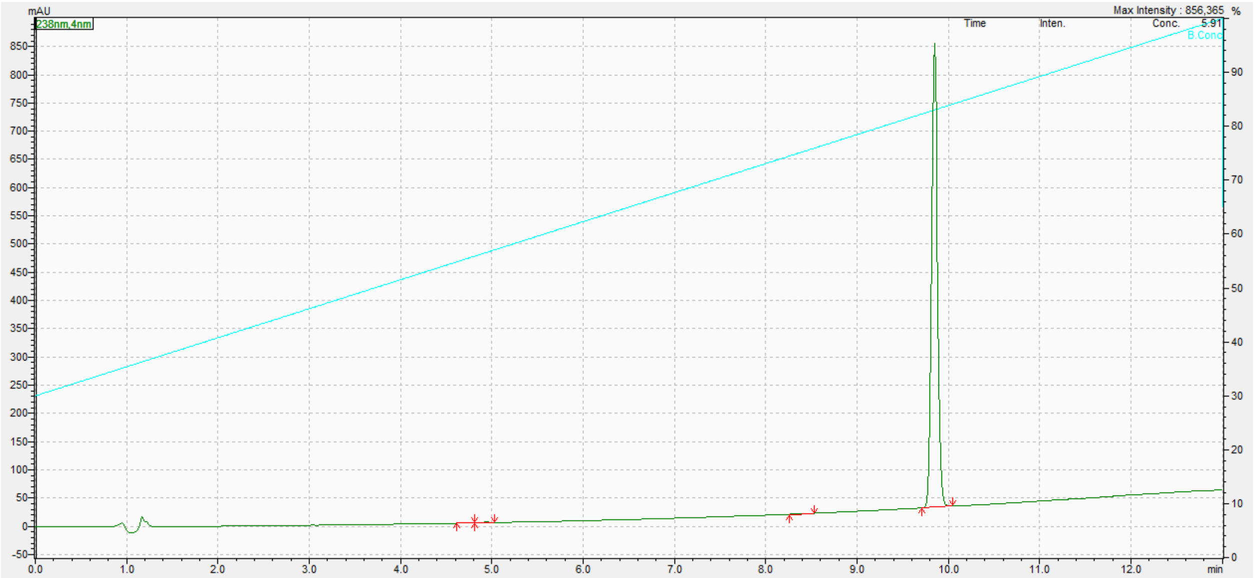

Figure S4. An example standard HPLC chromatogram of the solid (*S*)-**1·2** at 238 nm.

UV/Vis:

Spectra were recorded using a fiber-coupled Avantes Starline AvaSpec-2048 spectrometer, with an Avantes AvaLight-DHc lamp as the light source. These spectra were processed using Avasoft 8.7 software. A 1 cm quartz cuvette was used for all measurements.

1.3. **Batch Reaction Setup**

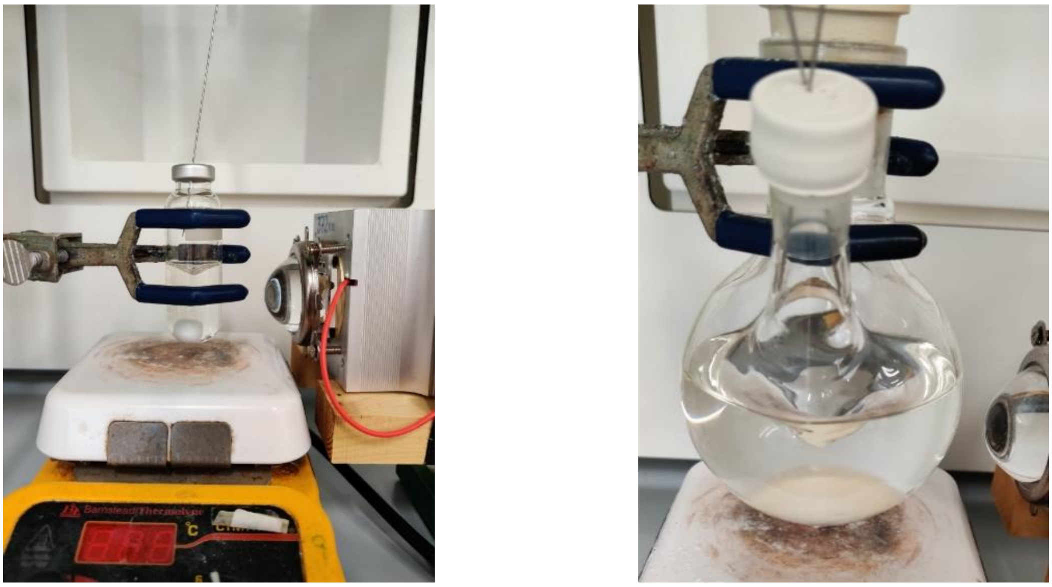

Figure S5. Photographs of small and larger scale reaction setup for racemization.

Light source: A 50 W (electrical power) LED wafer (372 nm wavelength), with focusing lens and fan/cooling block was situated ~5 cm from the reactor vessel.

1.4. **Batch Reaction Procedure (15 mmol)**

R-7-(*tert*-butyl)-6-chloro-2-(trifluoromethyl)-2*H*-chromene-3-carboxylic acid (2.52 g, 7.5 mmol) and ***S***-7-(*tert*-butyl)-6-chloro-2-(trifluoromethyl)-2*H*-chromene-3-carboxylic acid (2.52 g, 7.5 mmol) were added to a 250 mL three-necked flask. MeCN (150 mL) was added and the mixture was stirred, resulting in a fine suspension. The suspension was degassed with argon for 15 min (blown through from a balloon, with a needle positioned subsurface and an additional needle outlet to ensure gas flow). After degassing, L-phenylalaninol (1.09 g, 7.2 mmol) was added and the LEDs (input power 50 W) were turned on.

Samples were taken at regular intervals. After 29 h, a final sample was taken, then the slurry was filtered under vacuum and washed with MeCN (3 × 5 mL). The filter cake was further dried in a vacuum oven overnight at 40 °C to furnish the desired salt (*S*)-**1·2** (5.93 g, 81% yield, 99% ee) as a white solid.

1.5. **Flow Reaction Setup**

Reactor: Creaflow HANU HX 15-C276-CUB which consists of a Hastelloy baseplate composed of a series of cubic static mixing elements within a flow channel (530 mm × 60 mm × 45 mm size; 2 mm × 2 mm × 2 mm static mixing elements; 480 mm × 17 mm glass window; 2 mm × 2 mm channel dimensions; 15 mL internal volume).

Light source: An LED module (novaLIGHT FLED75 water-cooled high-performance LED array, Peschl Ultraviolet) equipped with 36 LEDs of 365 nm was placed on top of the reactor for UVA irradiation. Maximum current = 500 mA; radiative power at maximum current (setting “10”) = 32.4 W; width at 50% intensity = 9 nm.

Pumps: A Vapourtec SF-10 peristaltic pump, equipped with blue peristaltic tubing, was used as the metering pump. Oscillations were provided by a pulsator (ProMinent Beta/4 pump, PTFE/carbon pump head, customized by Creaflow), which was positioned between the peristaltic pump and the reactor. The frequency can be varied between 0.3 to 3 Hz (10 to 100%), while the amplitude can be tuned from 0.04 to 0.44 mL (<5 to 100%) per stroke.

Thermal regulation: The reaction path was cooled using tap water.

Inlet filter: To filter the inlet from the holdup vessel to the reactor, a Shimadzu suction filter (stainless steel, part # 228-45707-91) was used. Pore size = 20 µm, height = 1.8 cm, diameter = 1.2 cm, calculated surface area = 7.92 cm^2.

The reactor was stored under isopropanol between experiments for equipment compatibility reasons. Prior to reaction, the reactor was emptied of liquid by pumping argon through the system (argon balloon in feed vessel), then filled with MeCN and emptied in the same manner. In this way, the correct total volume of liquid could be ensured.

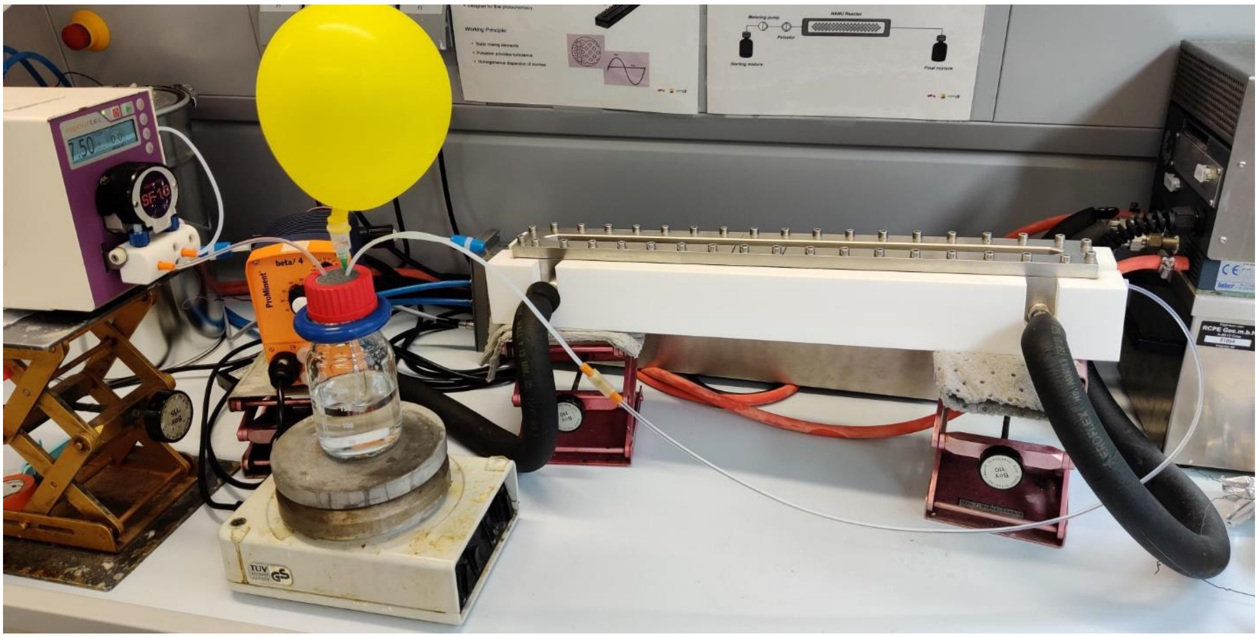

Figure S6. Photo of the HANU reactor setup, showing stirred batch vessel, dosing and pulsator pumps and photoreactor (without LEDs mounted).

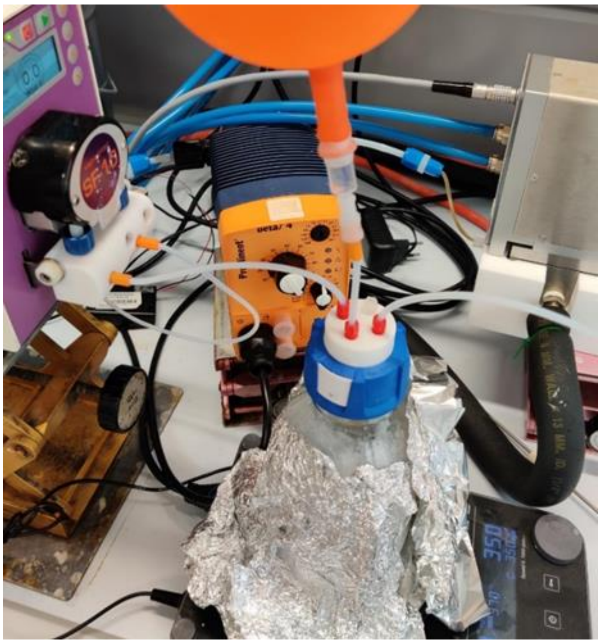

Figure S7. Photo of the foil-covered batch vessel used in the experimental setup, showing the Duran bottle lid, with input and output tubes, as well as an opening for an argon balloon.

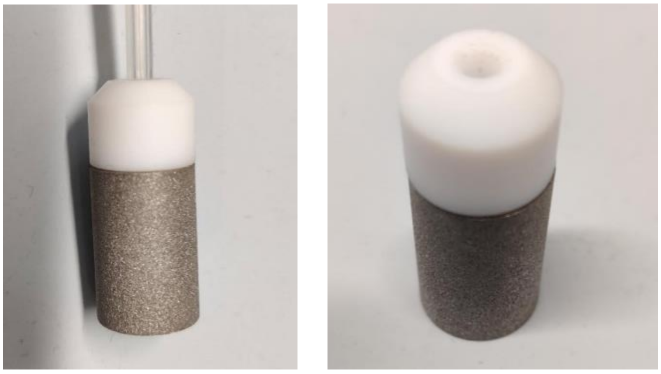

Figure S8. Photograph of inlet filter used in this study.

1.6. Representative Flow Reaction Procedure

R-7-(*tert*-butyl)-6-chloro-2-(trifluoromethyl)-2*H*-chromene-3-carboxylic acid (2.52 g, 7.5 mmol) and ***S***-7-(*tert*-butyl)-6-chloro-2-(trifluoromethyl)-2*H*-chromene-3-carboxylic acid (2.52 g, 7.5 mmol) were added to a 250 mL Duran flask (wrapped in foil). MeCN (150 mL) was added and the mixture was stirred, resulting in a fine suspension.

The metering pump was turned on at 10 mL/min and the pulsator pump to 100% amplitude, 50% frequency. In this manner, the reactor was filled with reaction solution and any gas bubbles were removed by tilting the pulsator pump and reactor at a positive angle (i.e. uphill flow direction).

The suspension was degassed with argon for 15 min (blown through from a balloon, with a needle positioned subsurface and an outlet in the bottle cap to ensure gas flow). After degassing, L-phenylalaninol (1.09 g, 7.2 mmol) was added and the LEDs (36 × 365 nm LEDs, total output power of 32.4 W) were turned on at 80% intensity.

After 30 min, a sample was taken and a further portion of L-phenylalaninol (1.09 g, 7.2 mmol) was added. After a further 30 min, a final portion of L-phenylalaninol (0.55 g, 3.6 mmol) was added.

Further samples were taken at 2 h and 4 h after switching on the LEDs.

After 6 h, a final sample was taken, then the reaction mixture was pushed out of the reactor by simply pumping gas through the reactor. The reactor was inclined at a negative angle (i.e. downhill flow) to ensure that as much liquid as possible was removed.

The collected slurry was filtered under vacuum and washed with MeCN (3 × 5 mL). The filter cake was further dried in a vacuum oven overnight at 40 °C to furnish the desired salt (*S*)-**1·2** as a white solid.

1.7. Sampling Procedure and Sample Preparation

Using a 1 mL disposable syringe, a homogeneous sample of liquid and solid phase (~0.2 mL) was taken up.

Liquid sample:

The sample was filtered through a syringe filter (0.45 µm pore size, LLG Labware, article # 6.272 817), then diluted with EtOH (1 mL). This solution was passed through a Si-SCX-2 ion exchange cartridge (Silicycle, product #: SPE-R51230B-01C). 1 µL of the solution was injected to the chiral HPLC. Standard HPLC used a 3 µL injection volume.

Solid sample:

MeCN (1 mL) was pushed through the syringe filter, to wash the solids of any remaining liquid phase. EtOH (1 mL) was pushed through the syringe filter to dissolve the solid phase. This EtOH was then passed through a Si-SCX-2 ion exchange cartridge (Silicycle, product #: SPE-R51230B-01C). 1 µL of the solution was injected to the chiral HPLC. Standard HPLC used a 3 µL injection volume.

2. UV/Vis Analysis of Starting Material

Prior to beginning resolution experiments, the starting material was examined by UV/vis spectroscopy, to gain an understanding of its absorbance behavior at 365 nm. The substrate shows an absorption maximum at 345 nm, with substantial absorbance still at 365 nm (**Figure S9**). The molar absorption coefficient at 365 nm was calculated to be 3727.8 L mol^{-1} cm^{-1} (**Figure S10**). This is relatively high for a substrate molecule and by Beer-Lambert calculations (0.1 M substrate concentration), >95% of light is absorbed within 0.04 mm of the vessel edge (**Figure S11**).

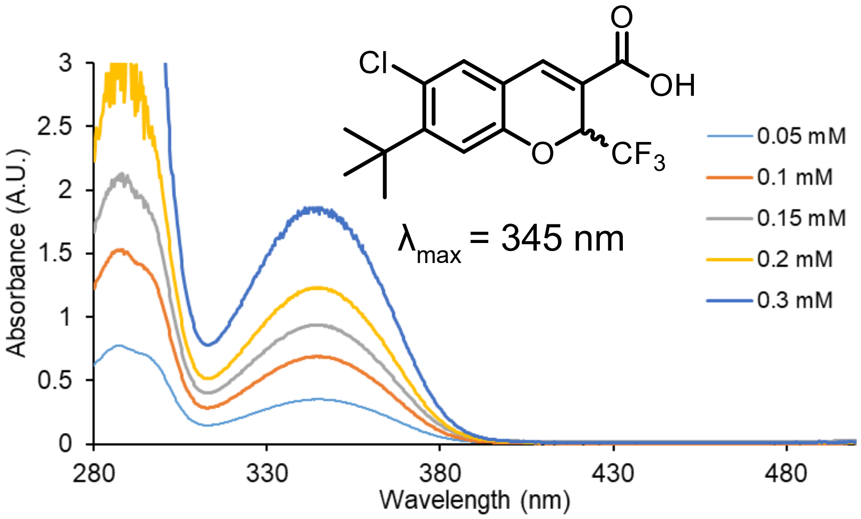

Figure S9. UV/Vis spectra of starting material at different concentrations (0.05-0.3 mM).

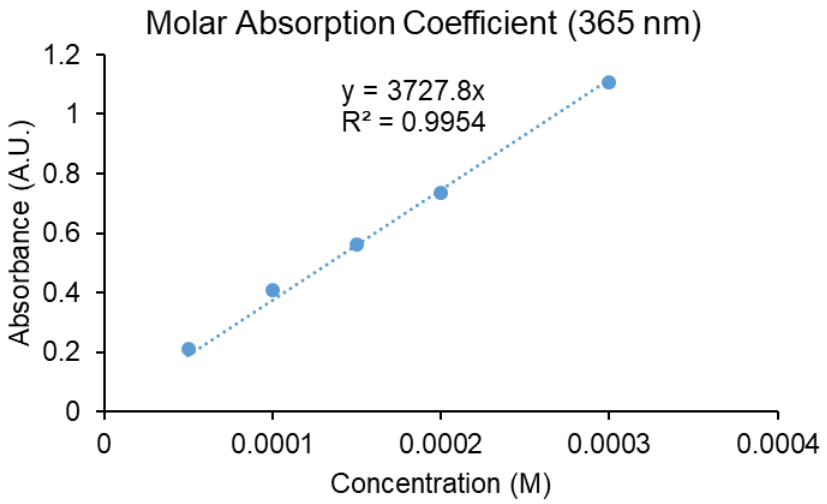

Figure S10. Molar absorption coefficient of the starting material at 365 nm, determined from the UV/vis spectra.

Using the Beer-Lambert law, the transmission (T) at different path lengths (i.e. distance from the reactor wall) can be calculated:

$$T = 10^{-\varepsilon c l} = 10^{(-3727.8 \times 0.1 \times \boldsymbol{l})}$$

Equation S1. The Beer-Lambert law, rearranged to calculated light transmission

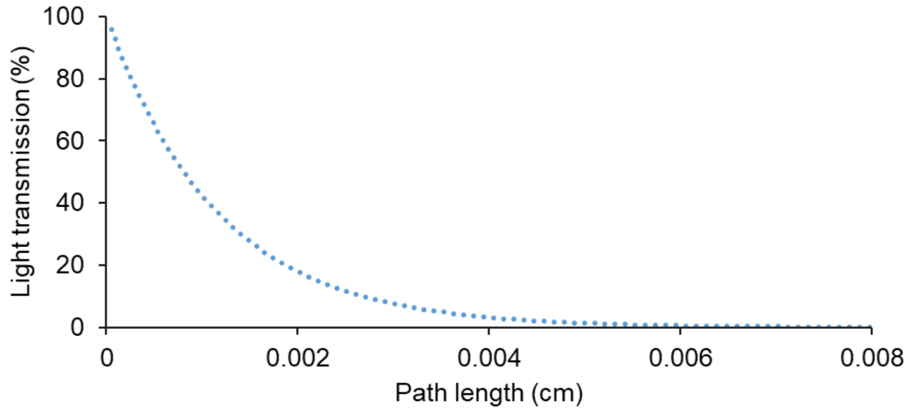

Figure S11. Theoretical light transmission under the reaction conditions (0.1 M substrate concentration).

3. ee Data of Liquid Phase

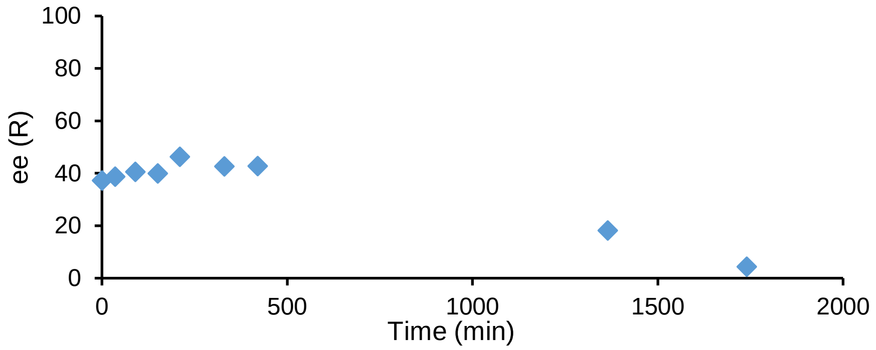

Figure S12. Evolution of the ee of the liquid phase, during large scale batch deracemization.

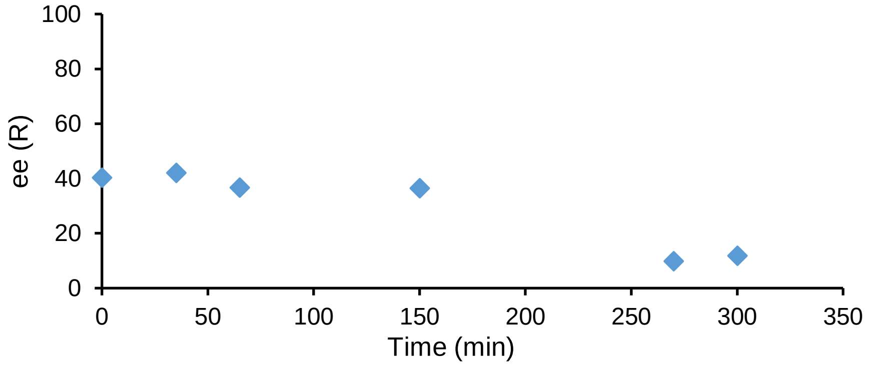

Figure S13. Evolution of the ee of the liquid phase, during flow deracemization, using initial conditions.

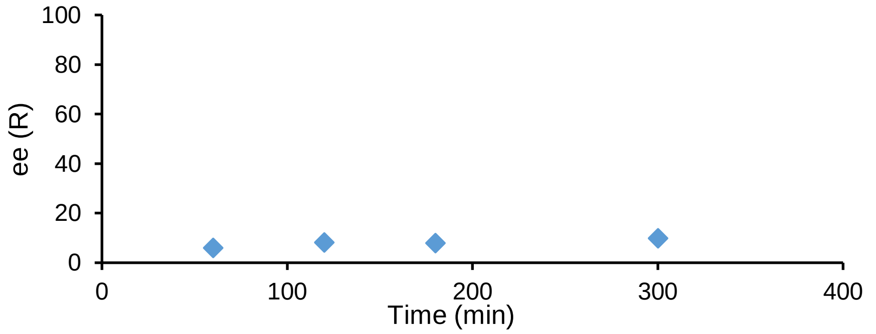

Figure S14. Evolution of the ee of the liquid phase, during flow deracemization, with portionwise addition of amine (0.2 equiv every 0.5 h).

4. DoE Data

4.8. DoE Setup

It was decided that a DoE study based on 4 factors should provide sufficient information to build useful models (**Table S1** Numerous different responses were measured (**Table S2**), in attempt to create models which describe the development of the resolution mixture over the duration of an experiment.

Table S1. Factors considered in full-factorial DoE study, with their minimum and maximum settings.

Factor	Description	Unit	Min.	Max.	Center
Light intensity	LED power setting between 0-10	-	6	10	8
Amine equiv	Stoichiometry of L-phenylalaninol used	Molar equivalents	1	1.4	1.2
Amine addition rate	Number of portions which the amine was divided into. A portion was added every 30 min. “5” provides the longest addition period, whereas “1” uses a single addition at the beginning of the resolution.	Portions	1	5	3
Scale	The quantity of 1 used. 10 mL of solvent per mmol were always used, to provide a constant concentration (e.g. 10 mmol requires 100 mL solvent, whilst 20 mmol requires 200 mL solvent)	mmol	10	20	15

Table S2. Responses considered in full-factorial DoE study. Note: a separate model was built for each response.

Factor	Description	Unit
Yield	Percentage quantity of theoretical mass, after resolution completion (6 h), filtration, washing and drying.	%
ee isolated	Measured ee of the isolated material.	%
ee 0.5 h	After 0.5 h of resolution, a sample was taken and the ee of the solid measured by chiral HPLC.	%
ee 2 h	After 2 h of resolution, a sample was taken and the ee of the solid measured by chiral HPLC.	%
ee 4 h	After 4 h of resolution, a sample was taken and the ee of the solid measured by chiral HPLC.	%
ee 6 h	After 6 h of resolution, a sample was taken and the ee of the solid measured by chiral HPLC.	%

By combinations of maximum and minimum levels of the factors (a two level design), an experimental design with 16 (2^4) experiments was laid out. Two center point experiments were also added, in order to check for linearity in responses and to test the reproducibility in the experimental setup. **Figure S15** shows a spatial representation of what a 3 factor design (2^3 experiments) would look like (this is difficult to visualize for a 4 factor design, since it would require a fourth "dimension").

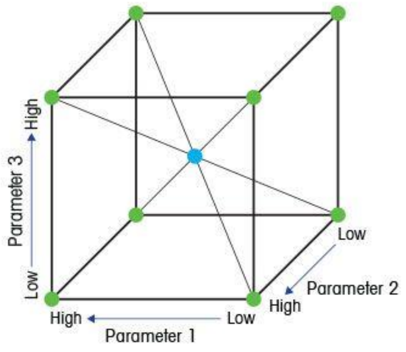

Figure S15. A pictorial representation of experiments carried out in a 3 factor full factorial DoE, i.e. high and low combinations for each factor, as well as "center point".

Table S3. All DoE experiments and results. Center point experiments are highlighted. **Note:** when an addition rate of 5 (slowest) was used, the 0.5 h sample did not contain solids, hence ee 0.5 h = 0 for all of these samples. **Note:** entries highlighted in grey were not included in their respective models.

	Factors					Responses						
Experiment Number	Light intensity	Amine equiv	Amine addn rate	Scale (mmol)		Yield	ee isolated		ee 0.5 h	ee 2 h	ee 4 h	ee 6 h
1	6	1	1	10		74.6	93.602		51.512	95.962	96.914	97.086
2	6	1.4	5	10		79	97.130		0	85.76	95.772	96.178
3	8	1.2	3	15		83.2	94.958		87.388	81.26	95.726	94.548
4	6	1	5	20		79.6	97.064		0	91.746	94.026	95.96
5	6	1	5	10		77.2	96.752		0	92.28	95.352	96.53
6	6	1.4	1	10		70.5	96.312		18.762	91.334	96.708	96.362
7	10	1	1	20		77.5	96.334		28.114	72.952	95.836	95.814
8	10	1.4	5	20		72.5	96.764		74.392	72.716	94.564	96.524
9	10	1	1	10		71.4	88.26		84.65	94.526	96.102	97.618
10	10	1	5	20		80.4	94.688		0	92.89	94.562	95.998
11	6	1	1	20		76.7	91.972		39.696	78.542	94.696	96.948
12	10	1.4	5	10		78	96.266		0	81.392	94.758	96.186
13	6	1.4	1	20		73	72.884		14.776	56.046	75.678	93.088
14	10	1	5	10		69.2	97.968		0	92.104	95.94	97.66
15	10	1.4	1	20		66.6	60.294		18.196	38.37	80.214	93.132
16	8	1.2	3	15		66.5	90.550		90.984	90.212	95.754	96.152
17	10	1.4	1	10		79.4	96.176		20.584	93.016	96.608	
18	6	1.4	5	20		76.9	97.712		0	56.662	87.004	94.696

4.9. **Analysis of DoE Results**

The DoE design and analysis was conducted using Modde Pro v12.1 (Sartorius AG). Models were fitted to maximize the following statistics, as shown in the "summary of fit" plot for each statistic (**Figure S16**):

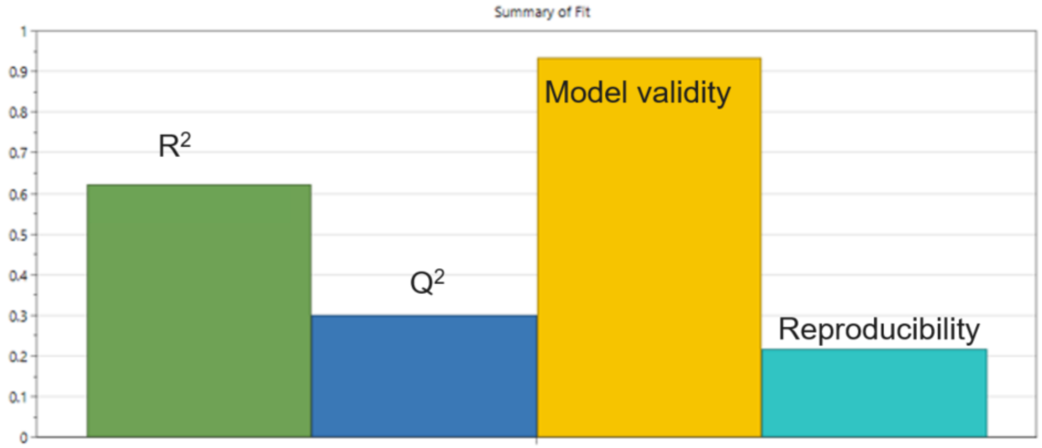

Figure S16. An example "summary of fit" plot, showing the four key statistics, explained below.

R^2

Shows the model fit. A model with R^2 of 1 is a perfect fit. A model with R^2 of 0.5 is a model with rather low significance.

Q^2 (most weight, due to "correction for overfitting")

Shows an estimate of the future prediction precision. Q^2 should be greater than 0.1 for a significant model and greater than 0.5 for a good model. The difference between R^2 and Q^2 should also be smaller than 0.3 for a good model. Q^2 is the best and most sensitive indicator.

Model validity (lowest weight, due to erroneous terms commonly causing low values)

A test of diverse model problems. A value less than 0.25 for *Model validity* indicates statistically significant model problems, such as the presence of outliers, an incorrect model, or a transformation problem. A low value here may also indicate that a term, such as an interaction or square is missing.

Reproducibility

The variation of the replicates compared to overall variability. The *Reproducibility* should be greater than 0.5

After conducting all experiments, models were generated for each of the responses. For models based on ee responses, a negative log transformation of the data was performed, in order to improve the data spread and enable better fitting.

4.9.1. Yield

Table S4. Statistics for "Yield" model. **Coeff SC** = value of coefficient. **Std. Err**. = standard error. **P** = probability of *incorrectly* assigning this coefficient value (closer to 0 is better). **Conf int (±)** = 95% confidence interval. **N** = number of samples in model. **DF** = degrees of freedom.

Yield	Coeff. SC	Std. Err.	P	Conf. int(±)
Constant	84.5848	2.30435	1.07E-13	5.02072
Amine equiv	-0.56489	0.784932	0.485511	1.71021
Addition rate	1.44375	0.802639	0.097239	1.74879
Scale	0.17451	0.794818	0.829904	1.73175
Equiv*Equiv	-9.5844	2.18324	0.000881	4.75684
Equiv*Scale	-2.37739	0.784932	0.01049	1.71021
N = 18	Q2 =	0.312		
DF = 12	R2 =	0.69		

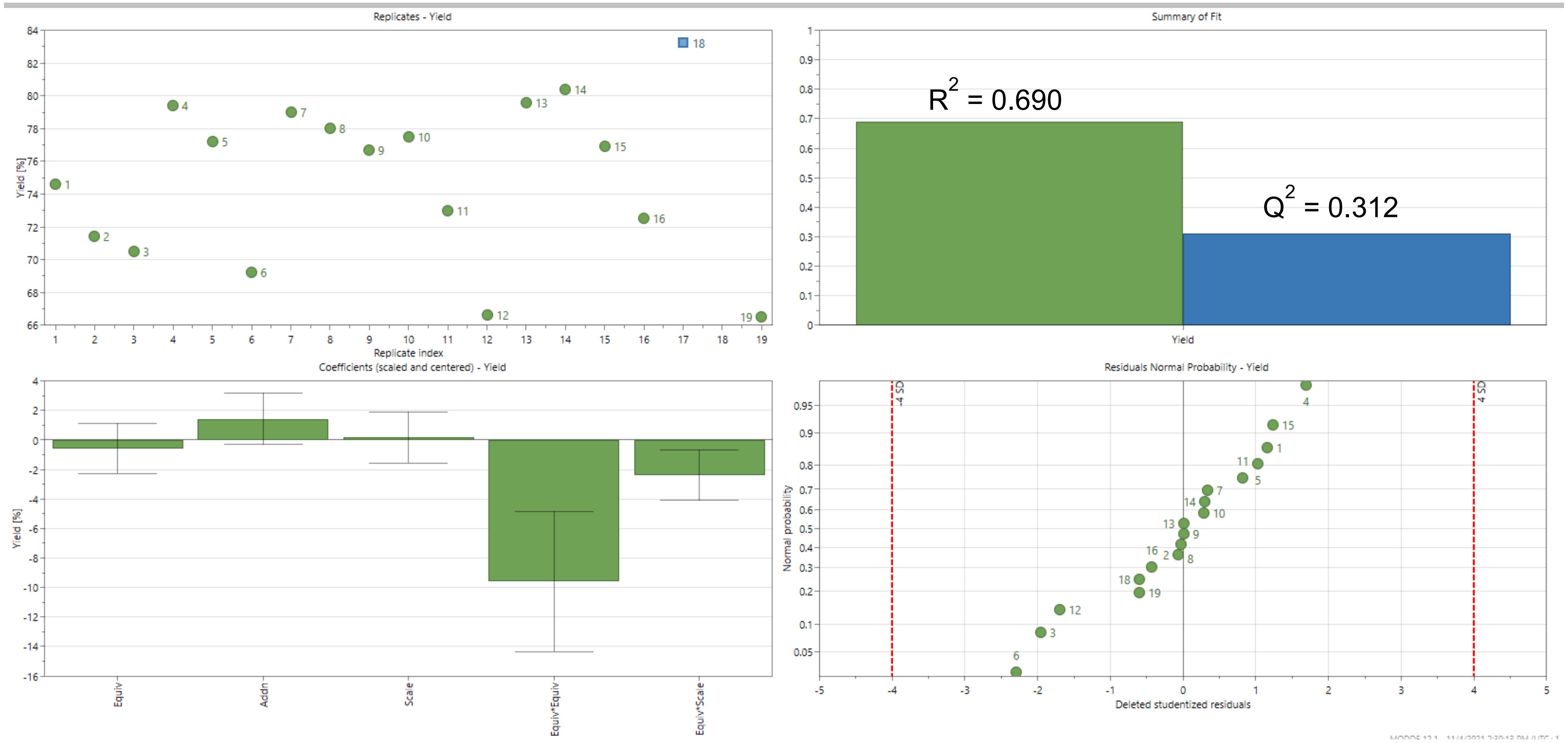

Figure S17. Model overview for "Yield" response.

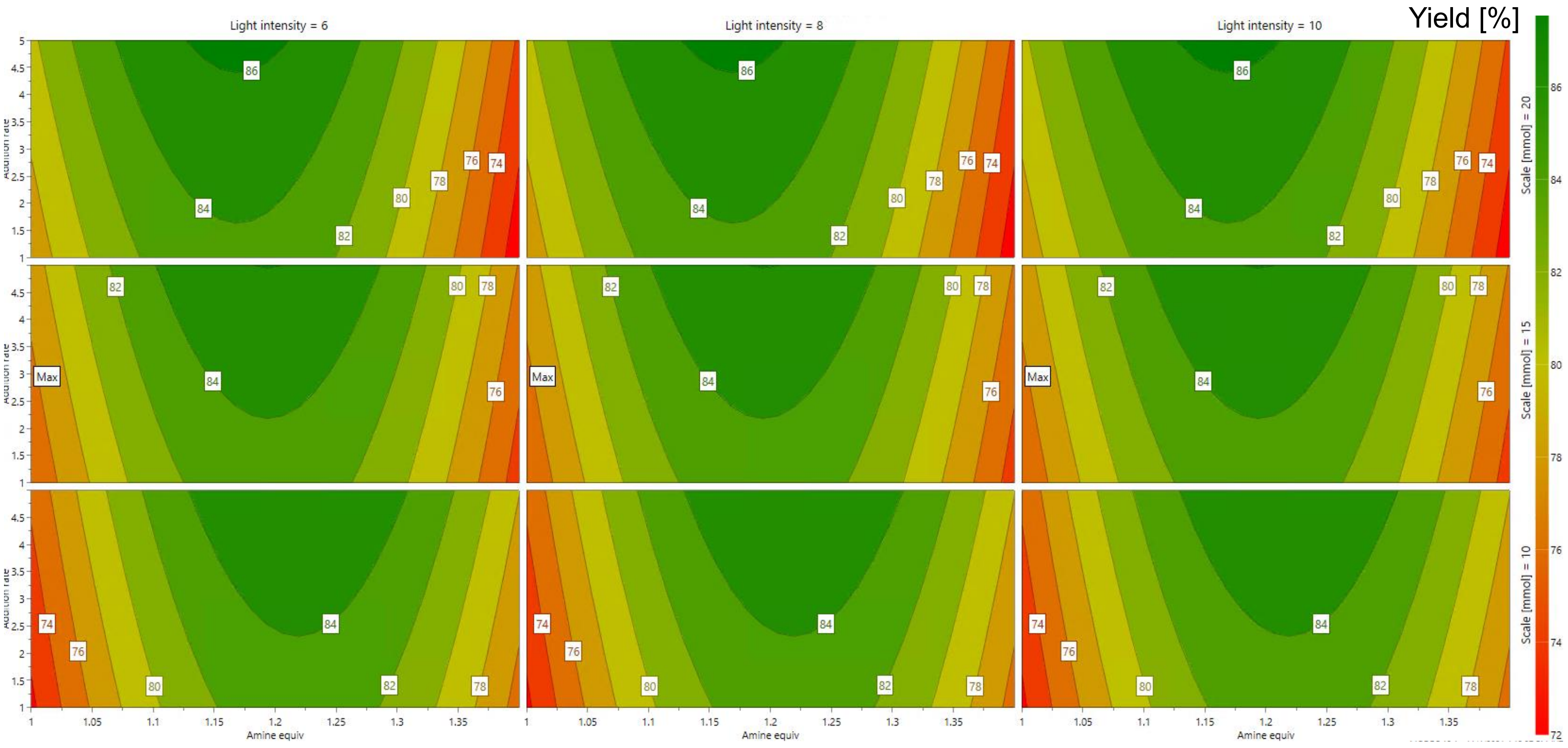

Figure S18. Contour plot for “Yield” response, showing response (color) as a function of 4 factors (axes).

4.9.2. ee Isolated

Table S5. Statistics for “ee Isolated” model. **Coeff SC** = value of coefficient. **Std. Err**. = standard error. **P** = probability of *incorrectly* assigning this coefficient value (closer to 0 is better). **Conf int (±)** = 95% confidence interval. **N** = number of samples in model. **DF** = degrees of freedom.

ee isolated	Coeff. SC	Std. Err.	P	Conf. int(±)
Constant	-0.81562	0.091787	1.26E-06	0.199985
Amine equiv	0.066948	0.04098	0.128271	0.089287
Addition rate	0.109288	0.043224	0.026496	0.094176
Addn*Addn	0.217317	0.099786	0.050087	0.217414
Equiv*Addn	-0.05721	0.043224	0.210328	0.094176
N = 17	Q2 =	0.302		
DF = 12	R2 =	0.622		

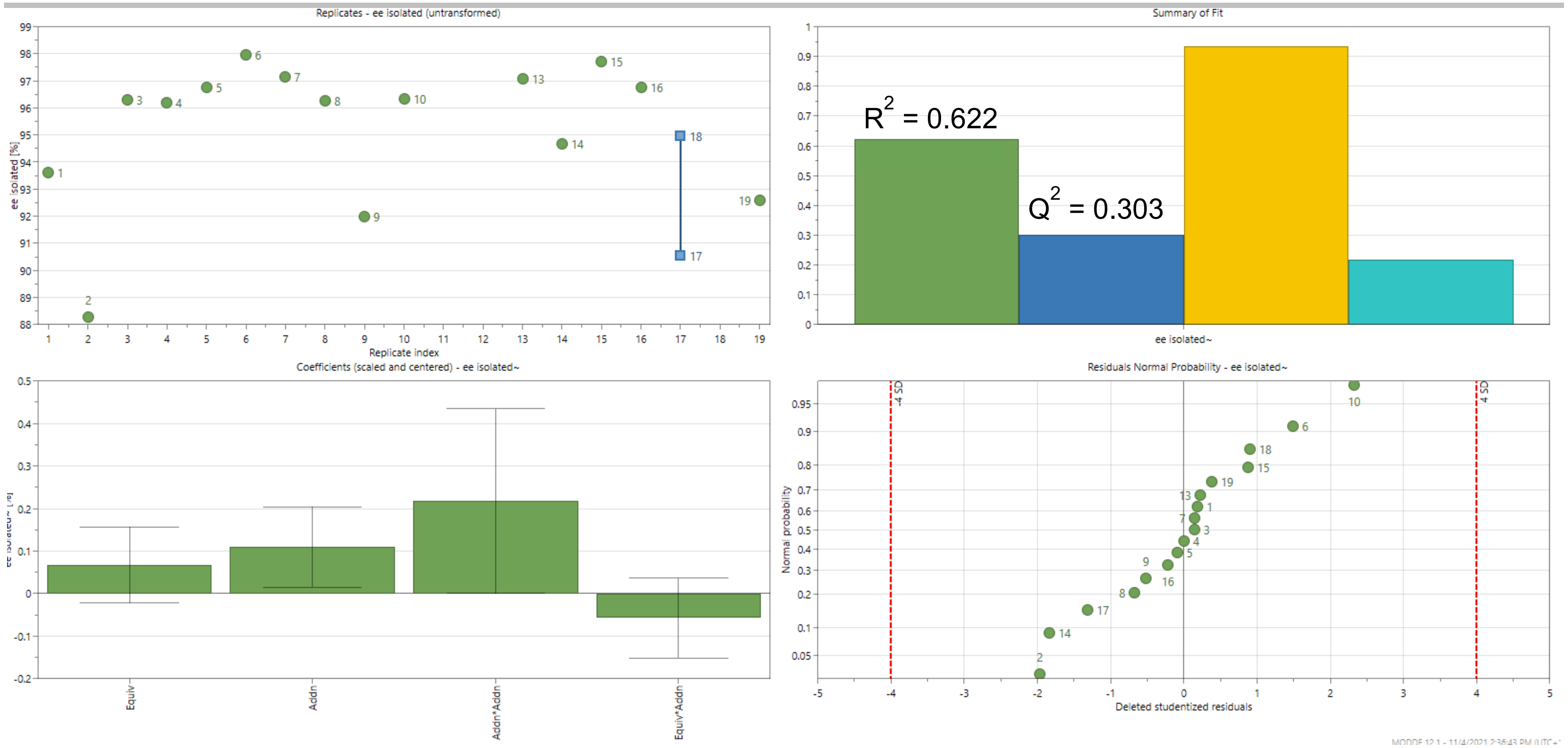

Figure S19. Model overview for “ee Isolated” response.

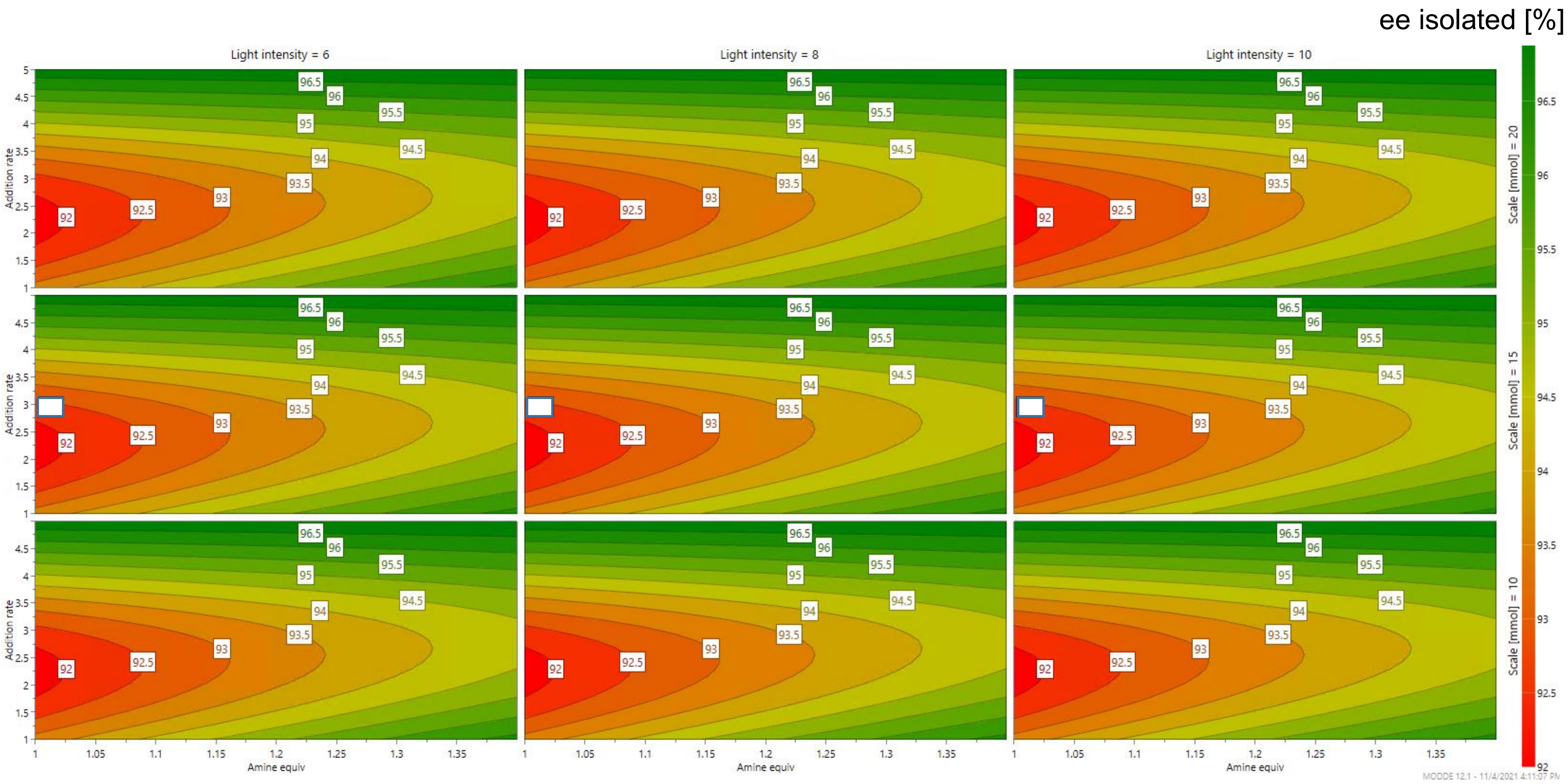

Figure S20. Contour plot for “ee Isolated” response, showing response (color) as a function of 4 factors (axes).

4.9.3. ee 0.5 h

Table S6. Statistics for “ee 0.5 h” model. **Coeff SC** = value of coefficient. **Std. Err**. = standard error. **P** = probability of *incorrectly* assigning this coefficient value (closer to 0 is better). **Conf int (±)** = 95% confidence interval. **N** = number of samples in model. **DF** = degrees of freedom.

ee 0.5 h	Coeff. SC	Std. Err.	P	Conf. int(±)
Constant	89.186	2.85477	6.31E-07	7.33844
Amine equiv	-12.2039	1.4513	0.00039	3.73069
Addition rate	28.7595	2.18247	4.49E-05	5.61024
Scale	-5.22614	1.58722	0.02165	4.08008
Scale*Scale	-30.0602	3.60684	0.000407	9.27169
Equiv*Scale	3.71556	1.4513	0.050636	3.73069
N = 11	Q2 =	0.976		
DF = 5	R2 =	0.99		

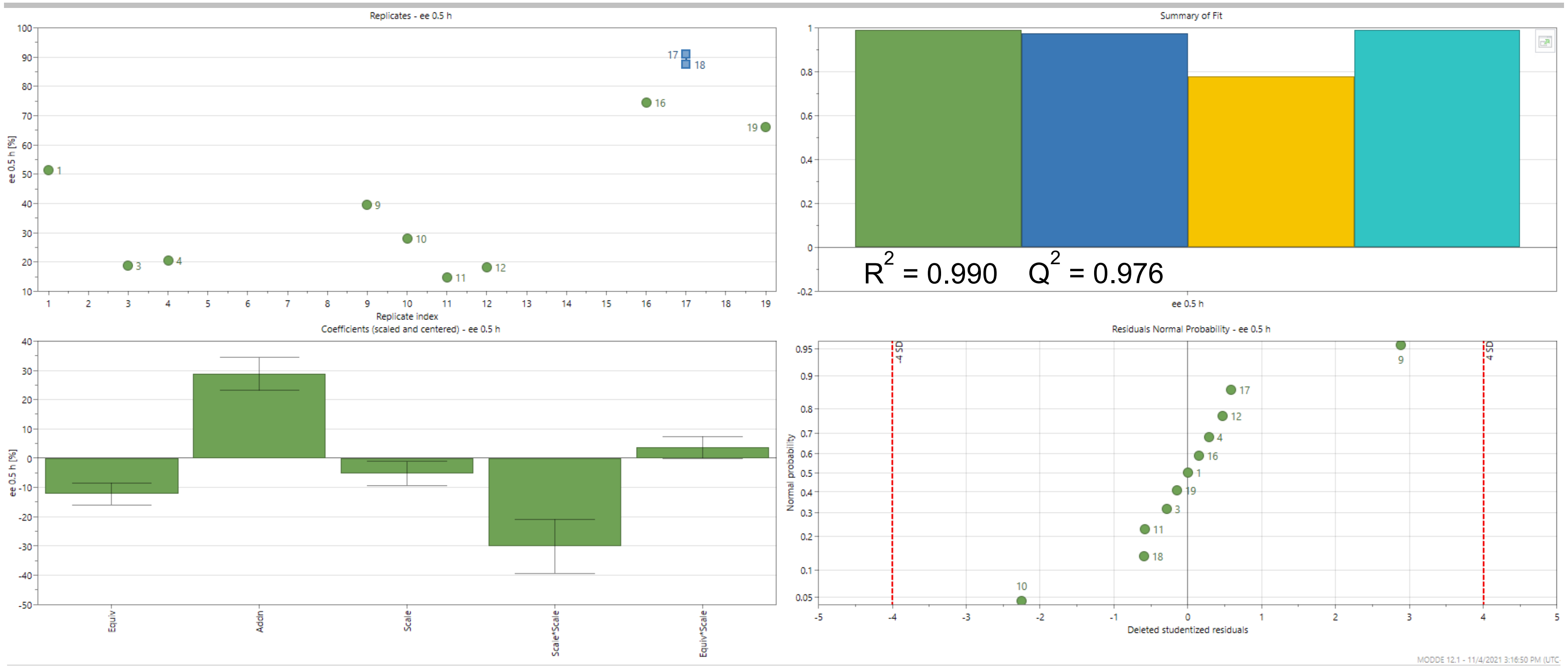

Figure S21. Model overview for “ee 0.5 h” response.

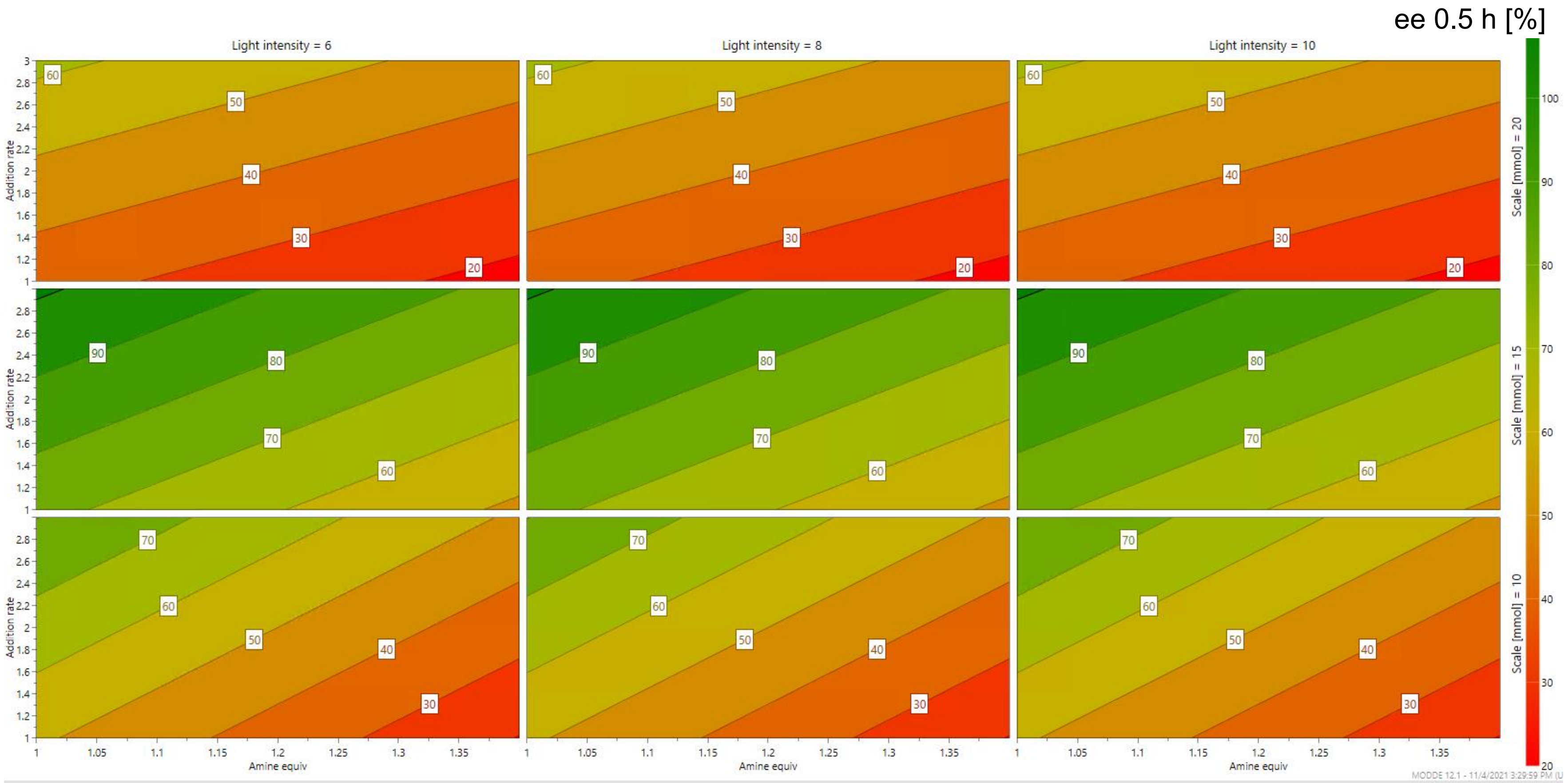

Figure S22. Contour plot for “ee 0.5 h” response, showing response (color) as a function of 4 factors (axes).

4.9.4. ee 2 h

Table S7. Statistics for “ee 2 h” model. **Coeff SC** = value of coefficient. **Std. Err**. = standard error. **P** = probability of *incorrectly* assigning this coefficient value (closer to 0 is better). **Conf int (±)** = 95% confidence interval. **N** = number of samples in model. **DF** = degrees of freedom.

ee 2 h~	Coeff. SC	Std. Err.	P	Conf. int(±)
Constant	-1.14476	0.025226	1.05E-15	0.054497
Amine equiv	-0.19339	0.025895	4.71E-06	0.055943
Addition rate	0.017085	0.027303	0.542305	0.058985
Scale	-0.22956	0.026687	1.00E-06	0.057653
Equiv*Scale	-0.05894	0.025895	0.04041	0.055943
Addn*Scale	0.152315	0.027303	8.94E-05	0.058985
N = 19	Q2 =	0.846		
DF = 13	R2 =	0.924		

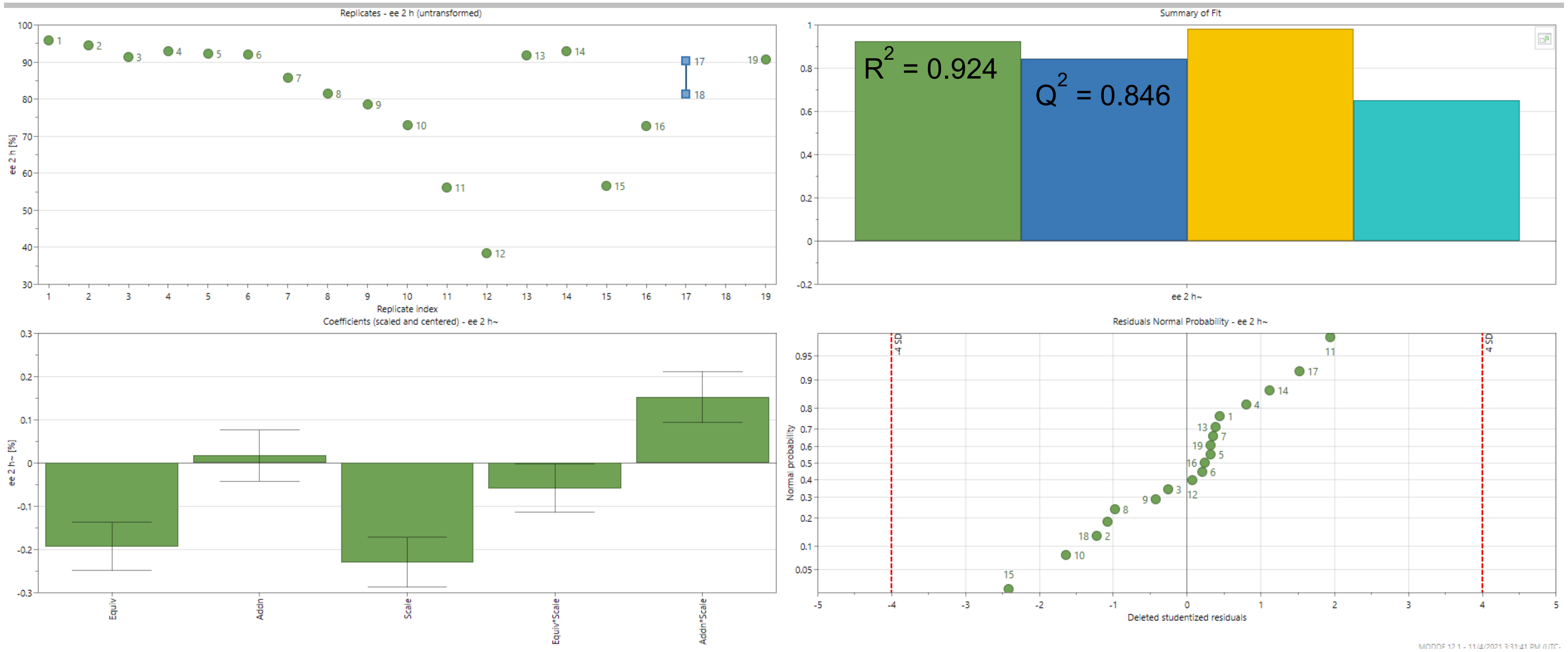

Figure S23. Model overview for “ee 2 h” response.

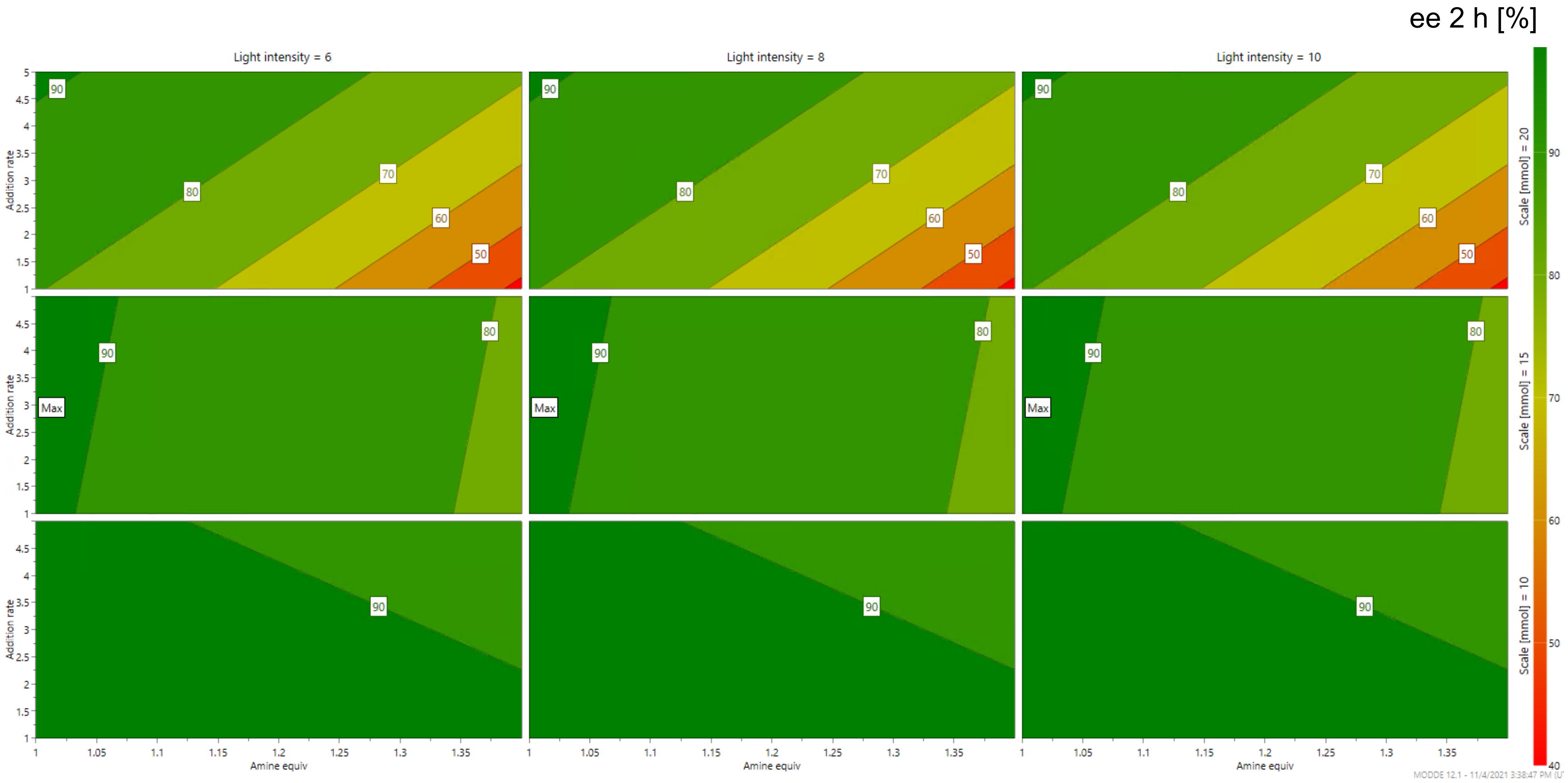

Figure S24. Contour plot for "ee 2 h" response, showing response (color) as a function of 4 factors (axes).

4.9.5. ee 4 h

Table S8. Statistics for "ee 4 h" model. **Coeff SC** = value of coefficient. **Std. Err**. = standard error. **P** = probability of *incorrectly* assigning this coefficient value (closer to 0 is better). **Conf int (±)** = 95% confidence interval. **N** = number of samples in model. **DF** = degrees of freedom.

ee 4 h~	Coeff. SC	Std. Err.	P	Conf. int(±)
Constant	-1.11085	0.010723	8.48E-18	0.023601
Amine equiv	-0.08595	0.005205	4.13E-09	0.011456
Addition rate	-0.01629	0.005308	0.010666	0.011682
Scale	-0.103	0.005248	6.54E-10	0.011551
Equiv*Equiv	-0.0911	0.010723	3.67E-06	0.023601
Equiv*Scale	-0.08431	0.005205	5.06E-09	0.011456
N = 17	Q2 =	0.948		
DF = 11	R2 =	0.983		

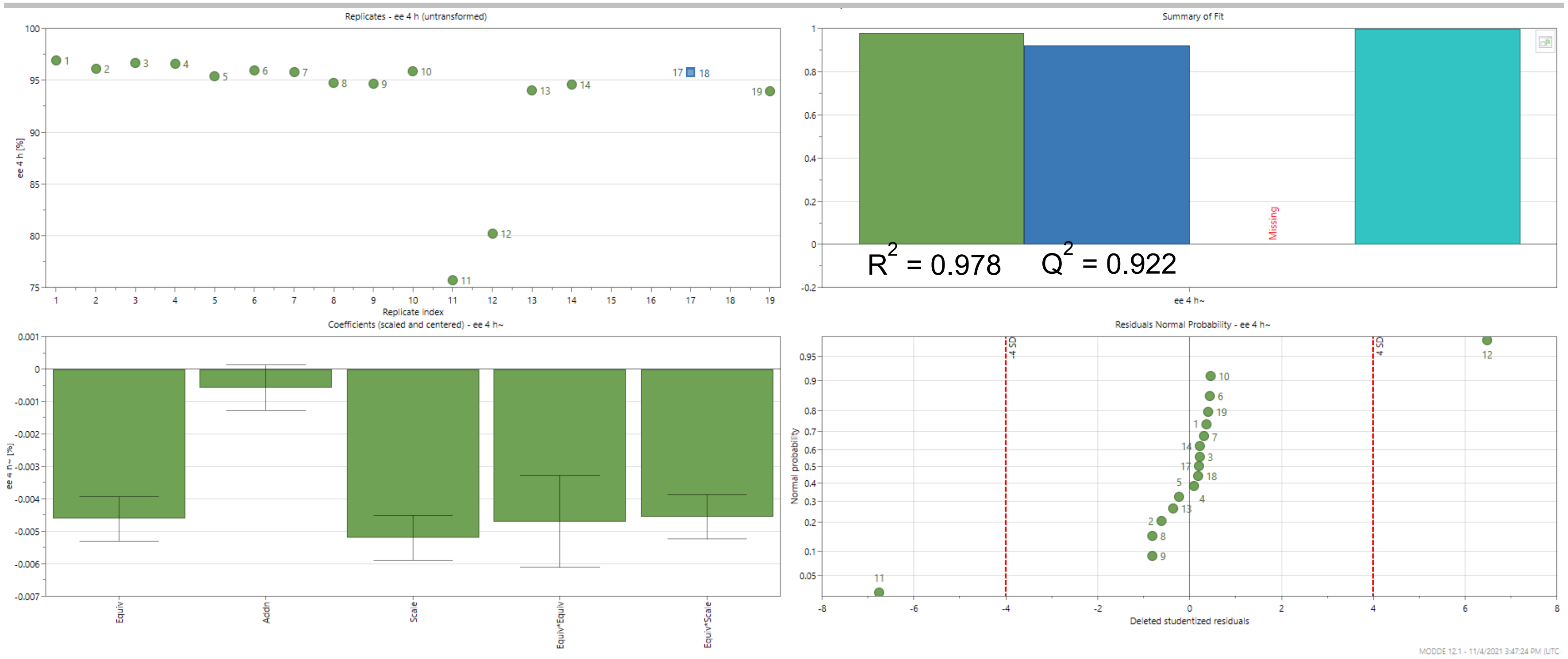

Figure S25. Model overview for “ee 4 h” response.

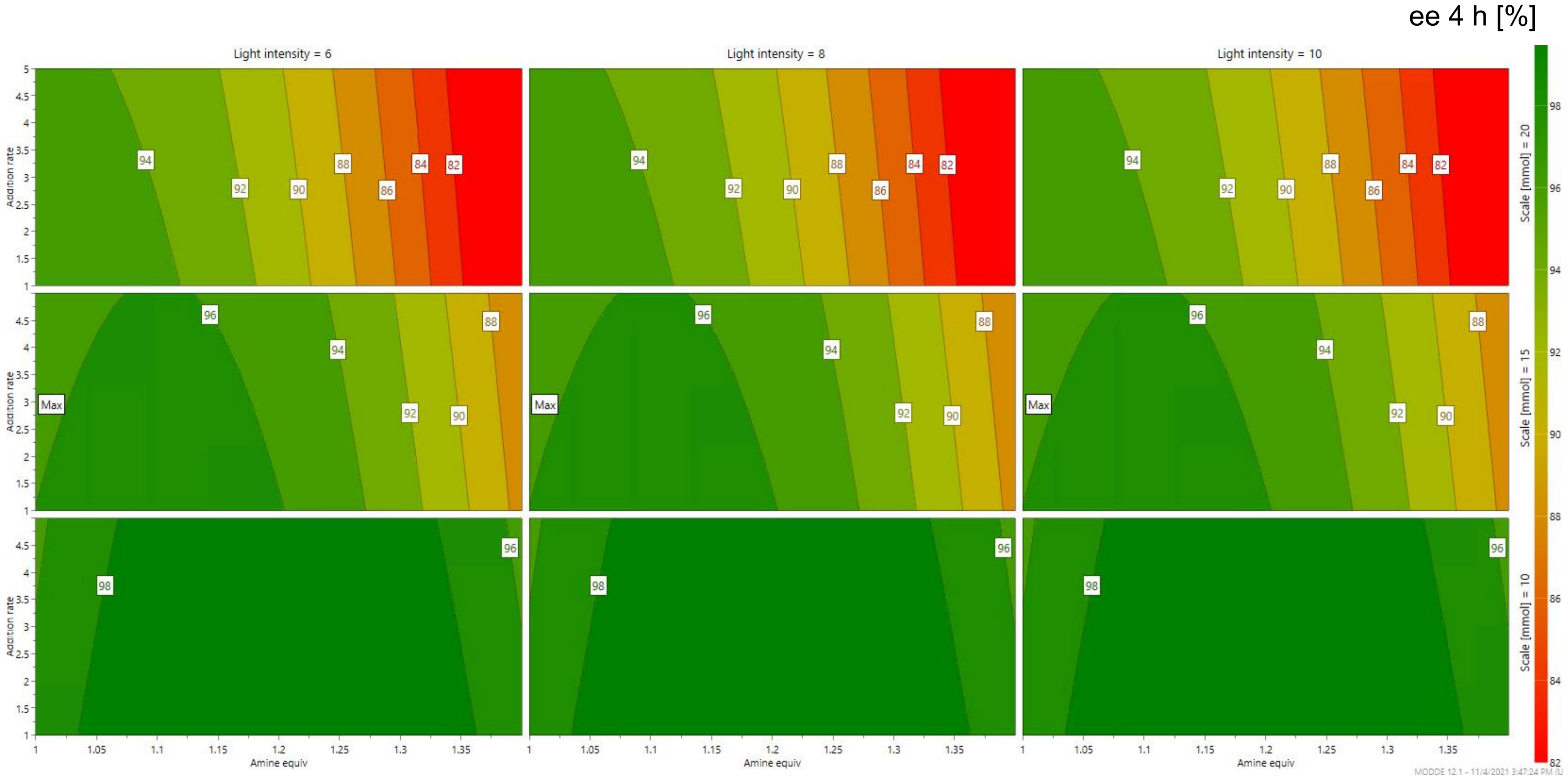

Figure S26. Contour plot for “ee 4 h” response, showing response (color) as a function of 4 factors (axes).

4.9.6. ee 6 h

Table S9. Statistics for “ee 6 h” model. **Coeff SC** = value of coefficient. **Std. Err**. = standard error. **P** = probability of *incorrectly* assigning this coefficient value (closer to 0 is better). **Conf int (±)** = 95% confidence interval. **N** = number of samples in model. **DF** = degrees of freedom.

ee 6 h~	Coeff. SC	Std. Err.	P	Conf. int(±)
Constant	-0.60121	0.069337	1.63E-06	0.151072
Amine equiv	-0.09523	0.028806	0.006272	0.062762
Addition rate	0.018336	0.03031	0.556471	0.066039
Scale	-0.10487	0.029573	0.004026	0.064434
Addn*Addn	0.156928	0.07494	0.058158	0.16328
Equiv*Addn	0.048612	0.03031	0.134723	0.066039
N = 18	Q2 =	0.365		
DF = 12	R2 =	0.72		

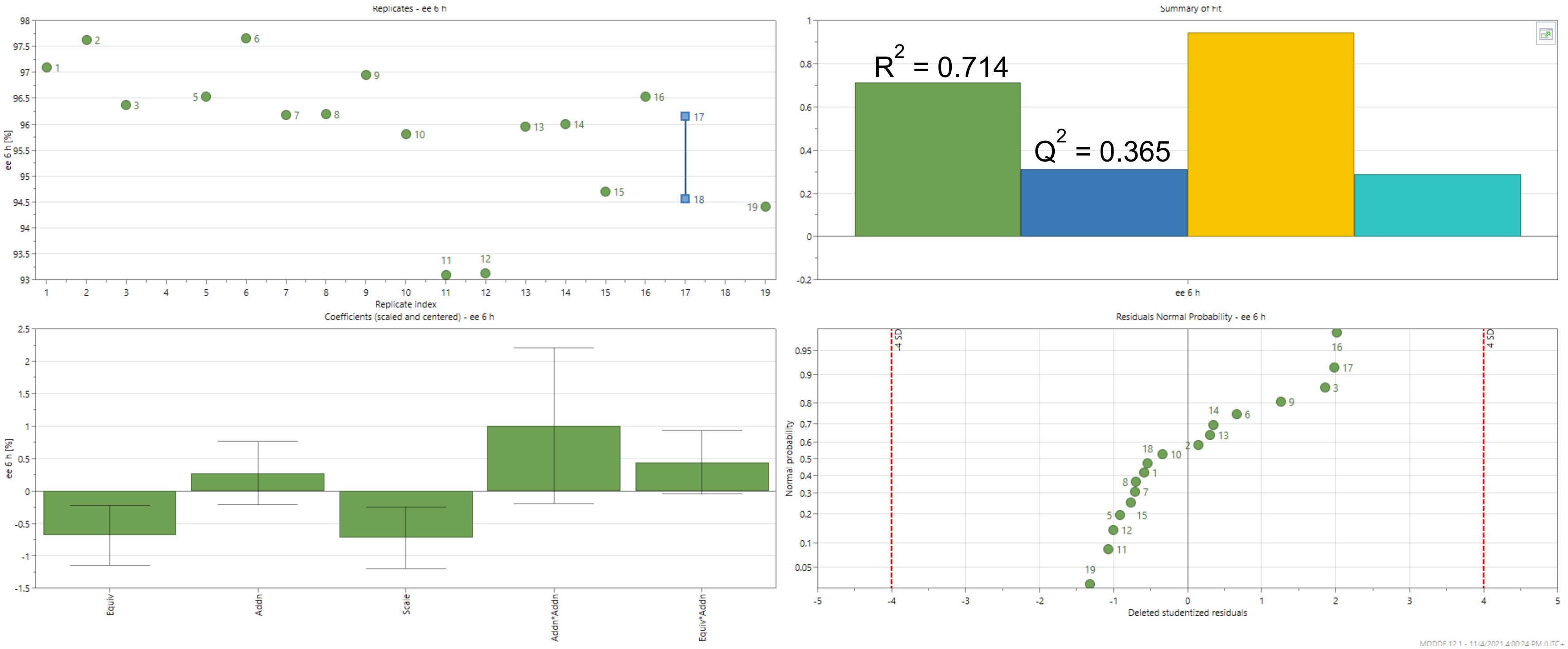

Figure S27. Model overview for “ee 6 h” response.

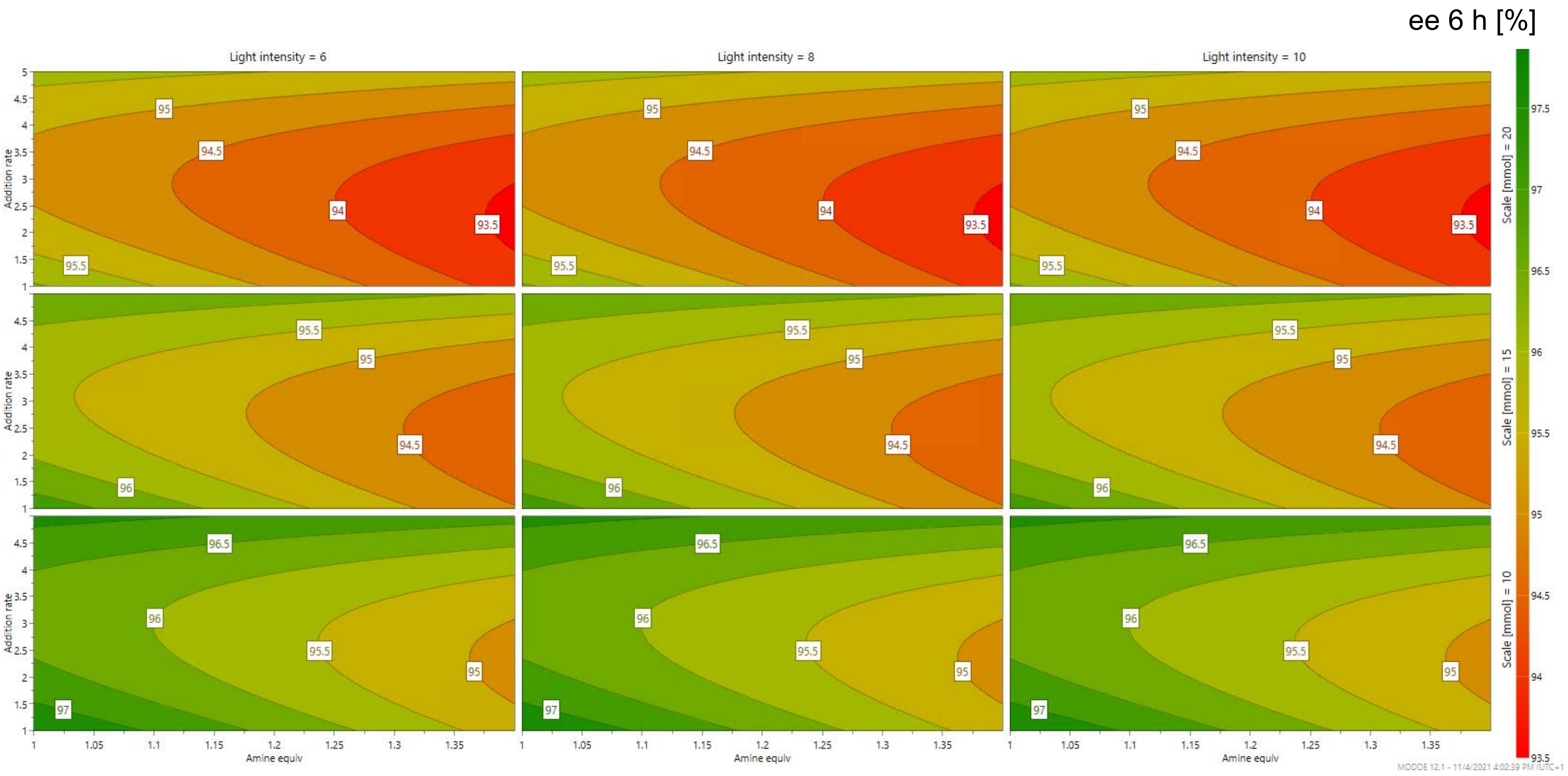

Figure S28. Contour plot for “ee 4 h” response, showing response (color) as a function of 4 factors (axes).

4.10. Validation Experiment

In order to validate the DoE models, an additional experiment was carried out, with the following factors, resulting in the corresponding responses (**Table S10**).

Table S10. Set points and results of the validation experiment, compared with the model predicted responses.

Factors					Responses					
Light intensity	Amine equiv	Amine addn rate	Scale (mmol)		Yield	ee isolated	ee 0.5 h	ee 2 h	ee 4 h	ee 6 h
8	0.9	3	20		66.5	92.6	66.2	90.8	94.0	94.4

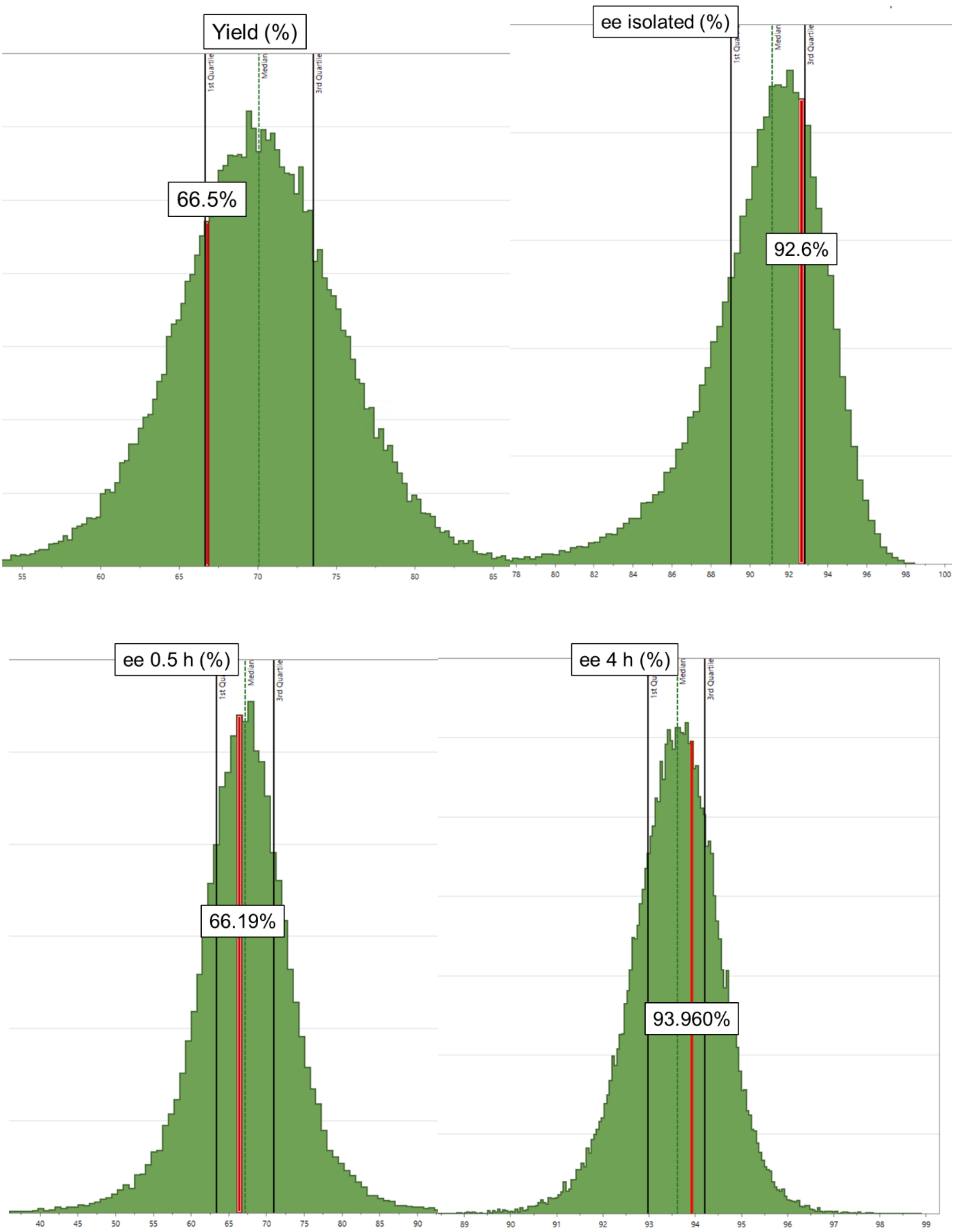

Figure S29. Plots of results from the validation experiment, versus the predicted values from each respective model. The black lines show the interquartile range, whereby the experimental results (red line) lie within these ranges.

4.11. **Final Experiment**

Based on the results of the DoE, an additional experiment was planned. The two most important models for the study (yield and ee at 4 h) were examined, and a “target” was added to each of the contour plots (**Figure S30**). This allows the corresponding set points to be predicted. The validation experiment was planned with the following set points:

1. Light intensity = 6
2. Amine equiv = 1.15
3. Amine addition = 3 portions
4. Scale = 20 mmol

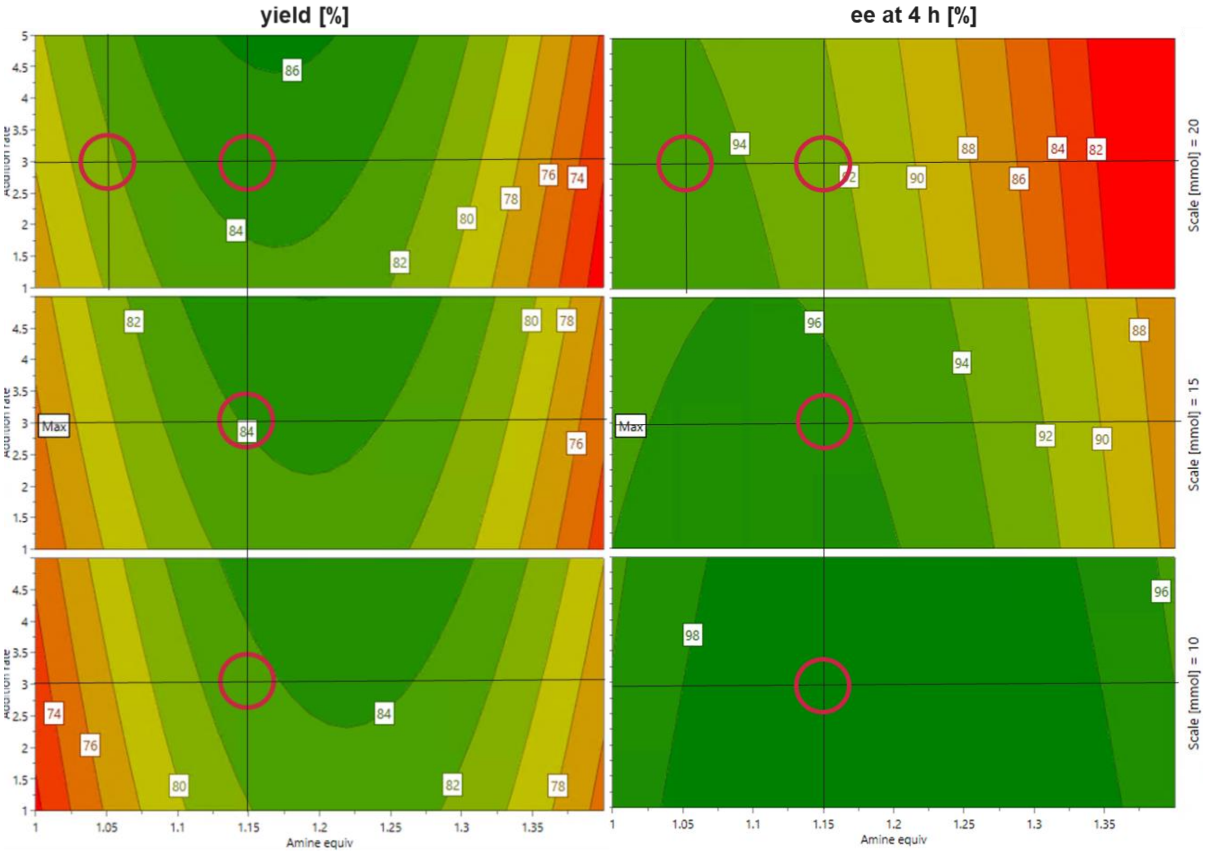

Figure S30. Contour plots for yield and ee 4 h, with a “target” response value, with the corresponding predicted required factor set points.

In attempt to improve the mixing in the batch vessel, the previously used Duran bottle was replaced with a glass jacketed vessel and an overhead stirrer (**Figure S31** and **Figure S32**). However, due to the small size of the overhead stirrer, the mixing proved to be insufficient in this setup.

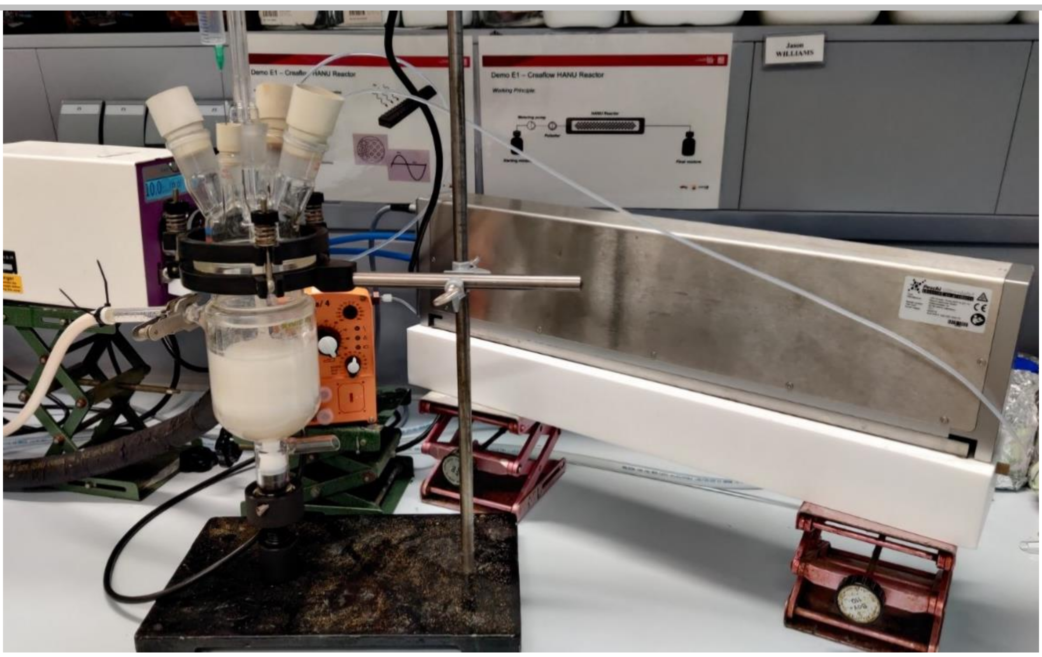

Figure S31. Photograph of the final experiment reaction setup, after addition of first amine portion.

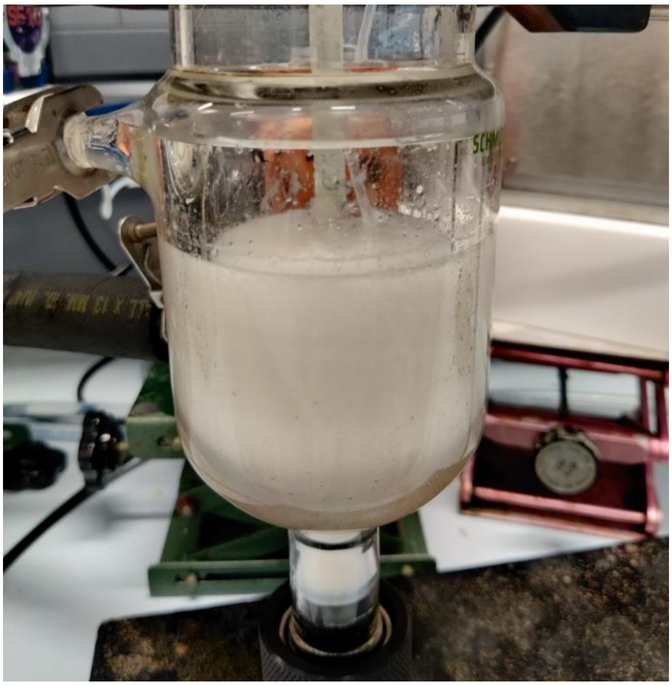

Figure S32. Photograph of the jacketed vessel and overhead stirrer, after addition of first amine portion.

The results of the final experiment are shown below (**Table S11**). These result are compared against the predicted values from the respective DoE models.

1. Yield: Lower than predicted, but remains on the higher end of the yields observed in all experiments thus far.
2. ee isolated: Lower than predicted. This can be attributed to the poor mixing, meaning that only a portion of reaction mixture was being circulated through the reactor.
3. ee 0.5 h: Higher than predicted.
4. ee 2 h: Lower than predicted and also lower than ee 0.5 h. This is likely because the mixture became significantly thicker (poorer mixing) after the addition of second and third portions of amine.
5. ee 4 h: Higher than predicted, however this may not be a representative sample, judging from the low ee after isolation.

Table S11. Set points and results of the validation experiment, compared with the model predicted responses.

**Factors**					**Responses**					
Light intensity	Amine equiv	Amine addn rate	Scale (mmol)		Yield	ee isolated		ee 0.5 h	ee 2 h	ee 4 h
6	1.15	3	20		81.6	61.4		86.5	73.9	96.0
**Predicted Responses**					84.9	93.2		56.0	79.5	93.3

5. Characterization Data

S-7-(*tert*-butyl)-6-chloro-2-(trifluoromethyl)-2*H*-chromene-3-carboxylic acid, (*S*)-2-amino-3-phenyl-1-propanol salt, (*S*)-**1·2**

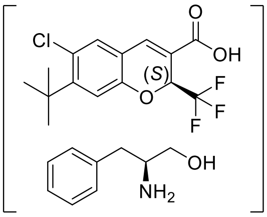

(*S*)-**1·2**

^{1}H NMR (300 MHz, Methanol-d$_4$) δ 7.43 – 7.17 (m, 7H), 7.01 (s, 1H), 5.84 (q, *J* = 7.3 Hz, 1H), 3.69 (dd, *J* = 11.4, 3.4 Hz, 1H), 3.57 – 3.39 (m, 2H), 2.94 (d, *J* = 7.3 Hz, 2H), 1.46 (s, 9H).

^{19}F NMR (471 MHz, Methanol-d_4) δ -79.63.

S-7-(*tert*-butyl)-6-chloro-2-(trifluoromethyl)-2*H*-chromene-3-carboxylic acid, (*S*)-**1**

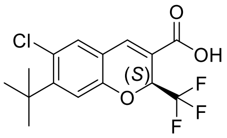

(*S*)-**1**

S-7-(*tert*-butyl)-6-chloro-2-(trifluoromethyl)-2*H*-chromene-3-carboxylic acid, (*S*)-2-amino-3-phenyl-1-propanol salt, (*S*)-**1·2** (100 mg) was dissolved in acetone (10 mL). Si-SCX-2 ion exchange silica (1.0 g) was added, then the mixture agitated and filtered. The resulting solution was transferred to a round-bottomed flask, and solvent removed on a rotary evaporator, to afford (*S*)-**1** as a white solid.

^{1}H NMR (300 MHz, Acetone-d$_6$) 7.86 (s, 1H), 7.50 (s, 1H), 7.11 (d, *J* = 0.8 Hz, 1H), 5.84 (q, *J* = 7.1 Hz, 1H), 1.48 (s, 9H).

^{19}F NMR (471 MHz, Acetone-d_6) δ -79.00.

NMR analysis is in agreement with previous reports.[S1]

6. References

[S1] J. S. Carter, M. G. Obukowicz, B. Devadas, J. J. Talley, D. L. Brown, M. J. Graneto, S. R. Bertenshaw, D. J. Rogier, S. R. Nagarajan, C. E. Hanau, et al., PCT Intl. Appl. US 006077850A, **2000**.

7. NMR Spectra

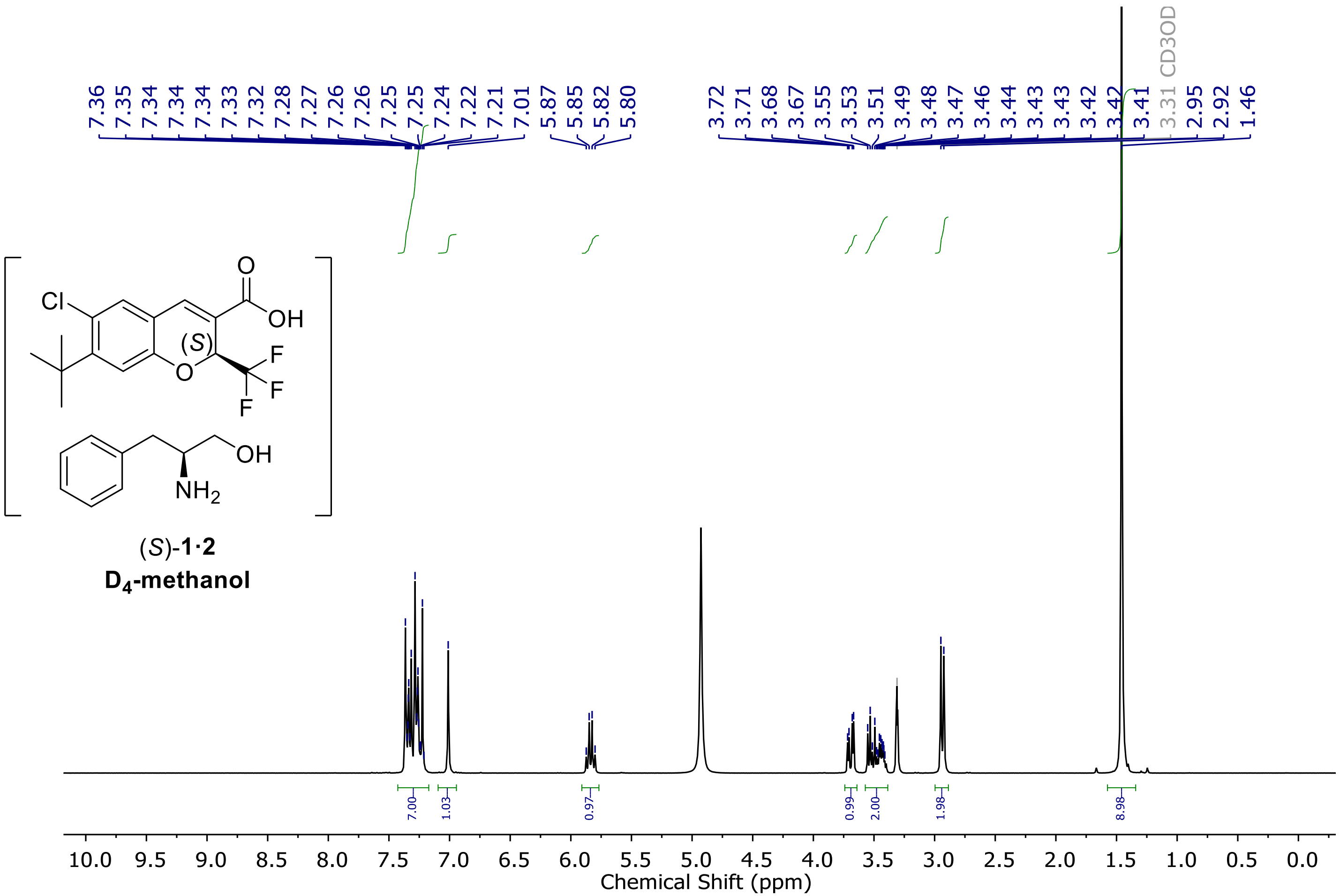

(*S*)-**1·2**

D_4-methanol

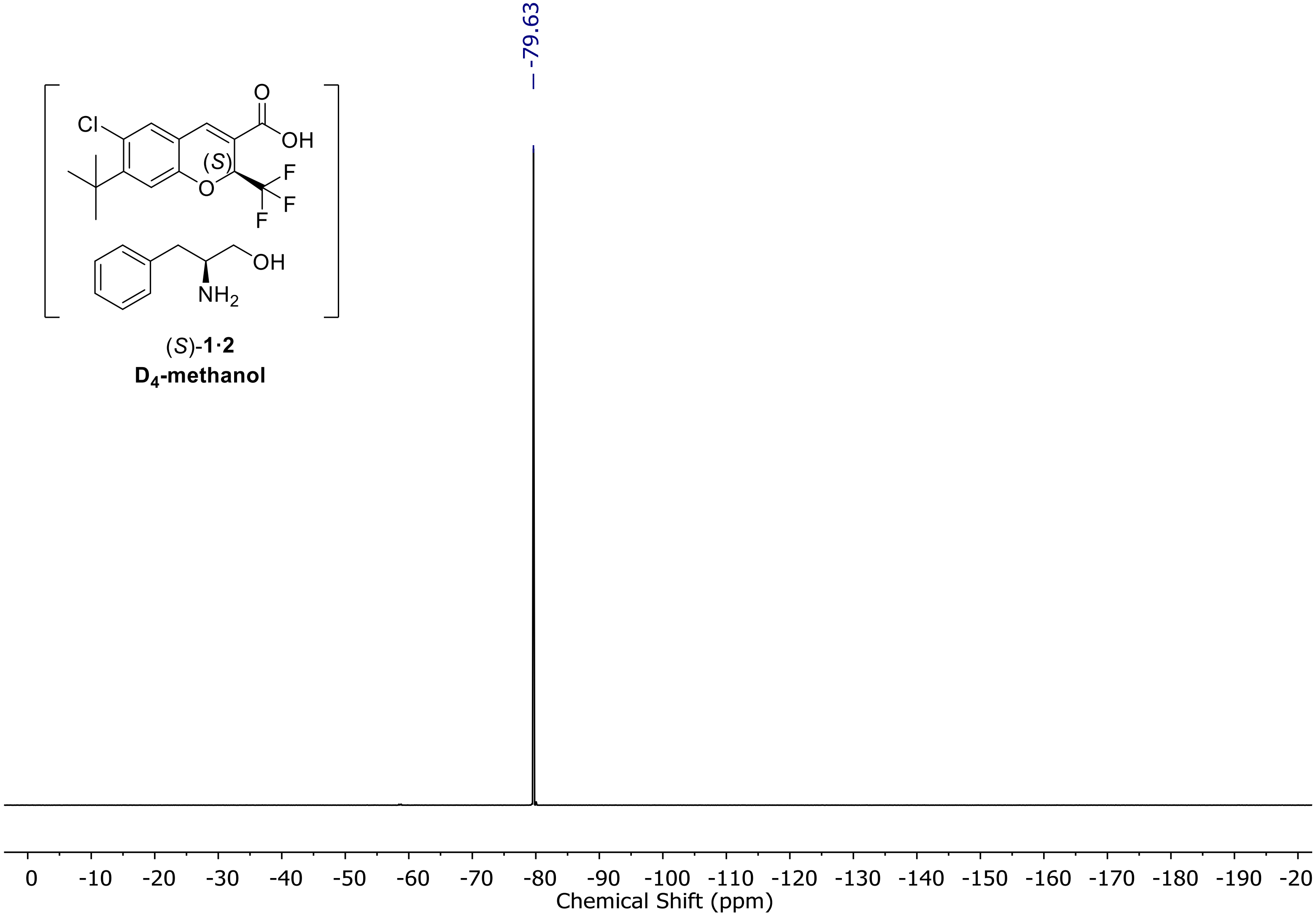
-79.63
Cl
O
OH
(S)
O
F
F
F
OH
NH2
(S)-1·2
D4-methanol
0 -10 -20 -30 -40 -50 -60 -70 -80 -90 -100 -110 -120 -130 -140 -150 -160 -170 -180 -190 -20
Chemical Shift (ppm)

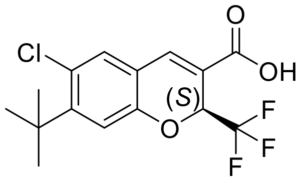

(*S*)-**1**
D_6-acetone

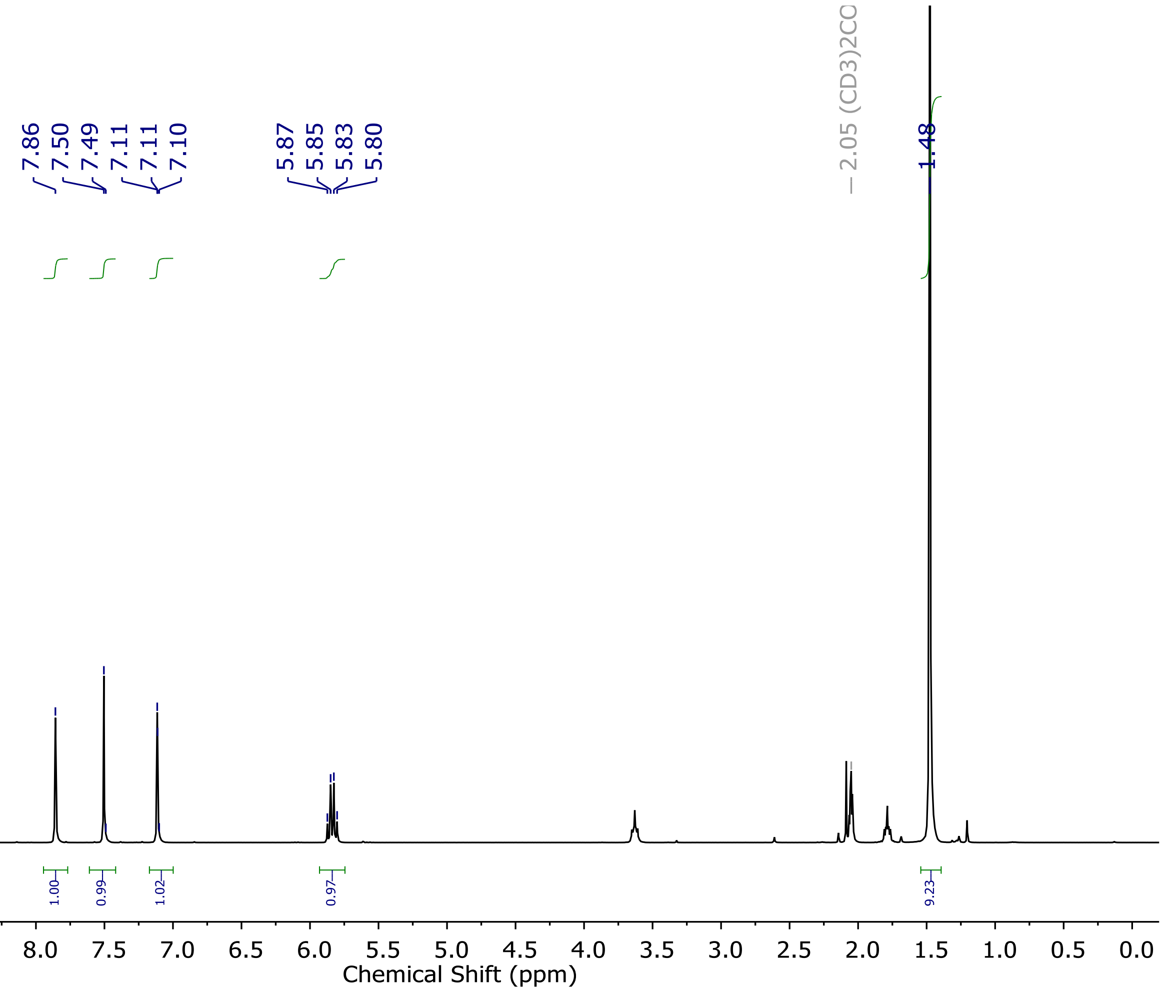

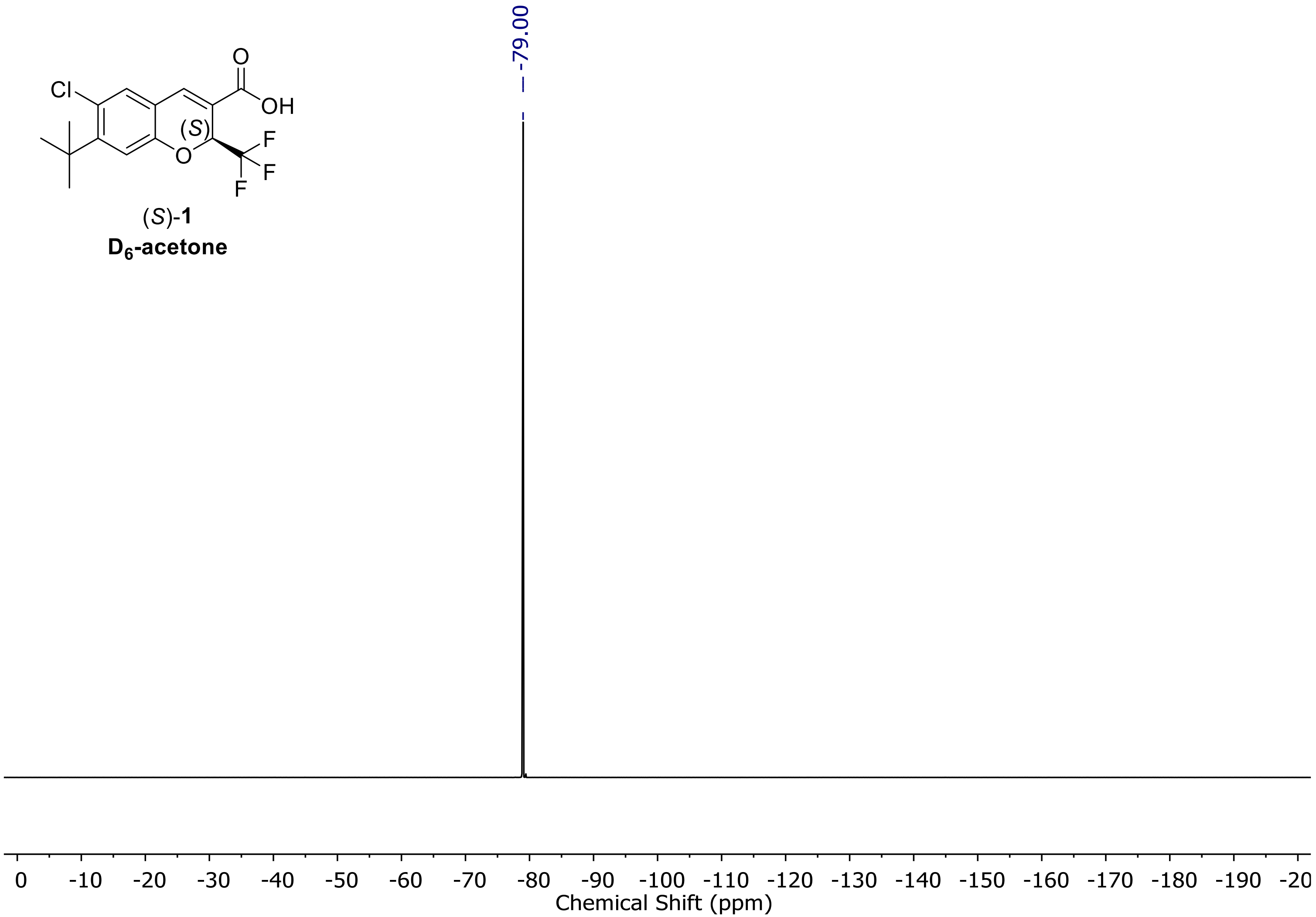
Cl
O
OH
(S)
O
F
F
F
(S)-1
D6-acetone
-79.00
0 -10 -20 -30 -40 -50 -60 -70 -80 -90 -100 -110 -120 -130 -140 -150 -160 -170 -180 -190 -20
Chemical Shift (ppm)
